# Cell Transplantation for Spinal Cord Injury: A Systematic Review

**DOI:** 10.1155/2013/786475

**Published:** 2013-01-15

**Authors:** Jun Li, Guilherme Lepski

**Affiliations:** ^1^Department of Neurosurgery, Eberhard Karls University, 72076 Tübingen, Germany; ^2^Department of Spine Surgery, The Affiliated Hospital of Luzhou Medical College, 646000 Luzhou, China; ^3^Division of Neurosurgery, Department of Neurology, Faculdade de Medicina, Universidade de São Paulo, Avnida Dr. Enéas de Carvalho Aguiar 255, 05403-000 São Paulo, SP, Brazil

## Abstract

Cell transplantation, as a therapeutic intervention for spinal cord injury (SCI), has been extensively studied by researchers in recent years. A number of different kinds of stem cells, neural progenitors, and glial cells have been tested in basic research, and most have been excluded from clinical studies because of a variety of reasons, including safety and efficacy. The signaling pathways, protein interactions, cellular behavior, and the differentiated fates of experimental cells have been studied *in vitro* in detail. Furthermore, the survival, proliferation, differentiation, and effects on promoting functional recovery of transplanted cells have also been examined in different animal SCI models. However, despite significant progress, a “bench to bedside” gap still exists. In this paper, we comprehensively cover publications in the field from the last years. The most commonly utilized cell lineages were covered in this paper and specific areas covered include survival of grafted cells, axonal regeneration and remyelination, sensory and motor functional recovery, and electrophysiological improvements. Finally we also review the literature on the *in vivo* tracking techniques for transplanted cells.

## 1. Introduction

During the last 20 years research on spinal cord injury (SCI) conducted in basic neuroscience research centers and neurology clinics has steadily increased. Researchers have investigated the issue from several angles, ranging from the design of novel therapeutic agents to elucidating the basic mechanisms underlying axon regeneration, remyelination, and inflammation; all with the aim of eventually promoting functional recovery in humans. Recent research has significantly advanced our understanding of SCI and has provided a few potential therapies. However, many questions remain unanswered and more continue to emerge. There has been a recent trend in the field to move towards combinatorial therapies, in an effort to synergize and boost the therapeutic effects of single therapies [1, 2]. Likewise there has also been increased interest in the use of pluripotent stem cells capable of differentiating into multiple cell types. Stem cell therapy for SCI is based on a strategy to treat the injuries and to restore lost functions by replacing lost or damaged cell populations [3].

Stem cells are several large series of immature and multipotential cells which can be found in all multicellular organisms. Self-renewal and multipotential differentiation are the two main characteristics of stem cells, and embryonic stem cells and adult stem cells are the two major categories [4, 5]. In 1903, Maximow proposed the hypothesis of stem cells at the congress of hematologic society in Berlin for the first of time [6]. Eighty nine years after the scientific use of the term of “*Stem Cell*”, neural stem cells were successfully cultured *in vitro* in 1992. These multipotential cells were generated from mammalian neural crest as neural spheres [7]. 

## 2. Spinal Cord Injury

Spinal cord injury (SCI) is caused by direct mechanical damage to the spinal cord that usually results in complete or incomplete loss of neural functions such as mobility and sensory function [8]. Motor vehicle accidents (40.4%), falls (27.9%), and acts of violence (15%) are the most frequent causes of SCI, and people with the average age of 40.7 years are most at risk [9]. The annual incidence of SCI is 40 cases per million population in the United States [10]. An estimated 12000 cases of paraplegia and quadriplegia are caused by SCI in the United States in each year, and approximately, 4000 patients die on the way to hospital and 1000 die during their hospitalization [11]. About 16% SCI patients have to live with life-long tetraplegia which is caused by high-level spinal cord injury [9]. 

The pathophysiological processes that underlie SCI comprise the primary and secondary phase of injury [10, 12]. The primary injury refers to the mechanical trauma to the spinal cord injury. In this phase, spinal cord tissue is disrupted by the force imparted by the primary injury mechanism. The most common injury mechanism is contusion of the spinal cord at the moment of injury and the prolonged compression caused by vertebral bony structures and soft tissues that have become dislodged [13]. During the injury process, the spinal cord might be hyper-bent, over-stretched, rotated, and lacerated [14], but the white matter is usually spared [15]. Although serious impairment of neural functions can be caused by the direct damage to the spinal cord tissue within the primary phase, the pathophysiological mechanisms involved in the secondary phase are an important determinant of the final extent of neurological deficits [8, 16].

Secondary damage occurs following the initial spinal cord trauma. The posttrauma inflammatory response plays a core role in the whole period of secondary phase after SCI though the modulation of a series of complex cellular and molecular interactions [17]. After spinal cord trauma, the blood-spinal-cord barrier, that protects and separates the spinal cord parenchyma from peripheral circulation, is broken down due to hemorrhage and local inflammation [18]. Increased production of chemokines (8–14 kDa polypeptides) and cytokines of the IL-1 family, which mediate the activation and recruitment of inflammatory cells t, is one of the triggers of SCI-induced inflammation [19]. The activation and recruitment of peripheral and resident inflammatory cells that include microglial cells, astrocytes, monocytes, T lymphocytes, and neutrophils further promotes the development of secondary damage following spinal cord injury [20]. The secondary phase of injury can be subdivided into the acute-phase (2 hours–2 days), the subacute phase (days–weeks), and the chronic phase (months–years) [13, 15, 16]. The pathophysiological changes that occur within these different phases are distinct. (1) Acute phase: edema, ischemia, haemorrhage, reactive oxygen species (ROS) production and lipid peroxidation, glutamate-mediated excitotoxicity, ionic dysregulation, blood-spinal-cord barrier permeability, inflammation, demyelination, neuronal cell death, and neurogenic shock. (2) Subacute phase: macrophage infiltration, microglial activity, astrocyte activity and scar formation, and initiation of neovascularization. (3) Chronic phase: Wallerian degeneration, glial scar maturation, cyst and syrinx formation, cavity formation, and schwannosis. The end of spontaneous post-SCI changes is identified as a pathophysiological phenomenon with solid glial scar formation, syrinx formation, and neuronal apoptosis. There is retraction and demyelination of spared axons which may induce permanent loss of sensorimotor functions that is unresponsive to treatment [21]. In order to select the best time-point for therapeutic cell transplantation, an understanding of the timeline of secondary damage cascades is important [22]. In order to promote functional recovery, stem cell transplantation must suppress the inflammatory response, inhibit neuronal apoptosis and necrosis, enhance neuronal regeneration, and promote axon regeneration and remyelination [23].

## 3. Cell Transplantation for Neural Regeneration

Cell transplantation may promote neural regeneration and rescue impaired neural function after spinal cord injury by means of (1) parasecreting permissive neurotrophic molecules at the lesion site to enhance the regenerative capacity; (2) providing a scaffold for the regeneration of axons; (3) replacing lost neurons and neural cells [24]. An early stem cell transplantation study in humans was reported as a one patient case report by a Korean research team in 2005. Multipotent adult stem cells from umbilical cord blood were directly injected into the lesion site of a SCI patient who had been nonstanding disabled for years, and the ability to walk was reported to be restored [25]. Then, a Chinese surgeon performed highly controversial experiment in China and claimed that about hundreds SCI patients who accepted direct injection of olfactory ensheathing cells, isolated from aborted fetuses, into the spinal cord were cured without complications [26, 27]. These studies were received with skepticism and general concern about the ethicality about the research [28, 29]. In recent years, the advancement of stem cell therapy for SCI has been encouraging and inspiring [30–32]. Nandoe Tewarie et al. explained the strategies of stem cells therapy for SCI [33], and several animal studies and clinical studies have demonstrated cellular regeneration and functional recovery using stem cells [30–32, 34, 35]. Although promising results for the treatment of subacute injury have been obtained, functional recovery still remains a challenge in the treatment of chronic injury [36]. 

## 4. Embryonic Stem Cells and Induced Pluripotent Stem Cells

Embryonic stem cells (ESCs) are a kind of pluripotent stem cells that can be derived from the inner cell mass of the early embryo [5]. Compared with the adult stem cells (ASCs) found in adults, ESCs are able to replicate indefinitely and to differentiate into all three primary germ layers cell lines and eventually generate all cell types in the body [37]. In contrast, the generated cell types from ASC differentiation were limited [38]. By introducing Sox2, Klf4, Oct3/4, and c-Myc, induced pluripotent stem cells (iPSCs) can be produced from cultured fibroblast with fewer ethical issues and reduced risk of immunological rejection and therefore may be more useful in clinical regenerative therapies [39]. According to the report from Miura et al., the iPS cells are capable of generating three main neural cell types *in vitro*, which are electrophysiologically functional neurons, astrocytes, and oligodendrocytes [40]. Recently, ESCs and iPSCs have been investigated to verify their therapeutic efficacy and their safety *in vivo* after SCI [41] (Table 1). Bottai et al. directly injected one million undifferentiated ESCs through the tail vein within 2 hours after the lesion. In this straightforward experiment, a significant improvement of BBB scores was confirmed in the experimental group when compared with the vehicle treated mice. In addition, an unexpected effect on the postinjury inflammatory response was also observed. The authors reported a greatly reduced number of invading macrophages and neutrophils. The authors speculated that the transplanted ESCs may improve lesion site preservation through this inflammation inhibition effect [42].

Since the ESCs and iPSCs have the capability to differentiate into all cell types, the most common strategy in rescuing the neural function after SCI is not the direct application of these cells, but the transplantation of various derived cell lines from ESCs or iPS cells. Several scientists have tried to generate neural progenitor/stem cells, motor neurons, oligodendrocyte progenitor cells, and olfactory ensheathing cells *in vitro*, and then transplant these cells into various animal models in order to verify the capability of neural function restoration *in vivo*. The derived cells that were injected into the animal models were restricted to one specific cell lineage, therefore reducing the risk of tumorigenesis when compared with directly applying ESCs or iPS cells [41]. Stem cell-derived neural stem/progenitor cells (NS/PCs) are currently considered a promising option of various cell replacement strategies for the treatment of spinal cord injury. However, these derived NS/PCs may possess variable characteristics depending on different derivation protocols. Using their own neurosphere-based culture system, firstly, Kumagai et al. [43] generated two different kinds of neurospheres, primary neurospheres (PNS) and passaged secondary neurospheres (SNS), these two kinds of neurospheres exhibit neurogenic and gliogenic potentials, respectively. Then, they transplanted PNS and SNS into rodent subacute SCI model. Interestingly, the positive results which included axonal growth promotion, remyelination, angiogenesis, and significant locomotor functional recovery were not obtained in the PNS group, but in the SNS group. This phenomenon might be induced by the neurotrophic parasecretion from gliogenic neurospheres transplantation, even though, this report still suggests that ESC-derived neurospheres are effective in promoting functional recovery after SCI *in vivo* [43]. Lowry et al. developed a novel coculture protocol with endothelial cells for treating mouse ESCs in the expansion phase with sonic hedgehog (Shh) and retinoic acid (RA) to generate motor neurons. The significant recovery of sensory and motor function in adult mouse SCI model was attained after transplantation of these motor neurons [44]. On perfecting the derivation protocol for the generation of consistent character NSCs from several different lines of ESCs and iPSCs, Koch et al. and Falk et al. presented a novel protocol which can produce a pure population of long-term self-renewing rosette-type ESC/iPSC-derived neural stem cells (lt-ESC/iPSC-NSCs) [113, 114]. This kind of lt-ESC/iPSC-NSCs exhibit consistent characteristics such as continuous expandability, stable neuronal and glial differentiation ability, and the capacity of generating functional mature neurons in monolayer culture. In order to verify the long-term ability of promoting functional recovery, Fujimoto et al. [45] transplanted lt-iPSC-NSCs into the lesion site of mouse. These grafted cells were observed not only to enhance remyelination and axon regeneration, but also to support the survival of endogenous neurons. More importantly, at the ninth week after transplantation, the previously attained motor function recovery was reduced significantly by means of the ablation of transplanted lt-iPSC-NSCs via the introduction of Diphtheria toxin. 

It is precisely because of the efficacy of neuronal regeneration and neuronal function promotion, a variety of ESC/iPSC-NSCs application strategies for SCI has been reported by several groups. The neural cell adhesion molecule L1 was thought to be able to promote the survival of grafted cells in the lesion site of central nervous system, and to favor axonal growth *in vivo* [46]. Based on this, Cui et al. transplanted L1-overexpressing substrate adherent embryonic stem cell-derived neural aggregates (SENAs) into a mouse SCI model. Eventually, an increased number of surviving cells, enhanced neuronal differentiation, reduced glial differentiation, and increased tyrosine hydroxylase expression was confirmed when compared with wild type SENAs transplanted group [47]. 

Neurogenins are a family of bHLH transcription factors involved in specifying neuronal differentiation. As a marker of neuronal differentiation, Neurogenin-2 (Ngn2) is essential for the development of CNS tissue as well, especially for the dentate gyrus [115]. By utilizing Ngn2 expressing ESC-derived NPCs, Perrin et al. and Shapiro et al. fully restored weight support and significantly improved functional motor recovery of rats after severe spinal cord compression injury. In addition, the expression of serotonin 5HT1A receptor, which is expressed in the raphespinal tract which plays a major role in locomotion and is particularly affected after SCI, was partially restored [48, 116]. In another report, Hatami et al. injected human embryonic stem cell-derived NPCs (hESC-NPCs) with collagen scaffolds into hemisection rat model. As reported, the grafted hESC-NPCs successfully differentiated into neurons and glia *in vivo*, and promoted hindlimb locomotor recovery and sensory responses with observed migration of transplanted stem cells toward the lesion site [49]. The collagen scaffolds were believed to support the survival of transplanted cells at the initial phase of transplantation *in vivo*. In a recent report, cotransplantation of hESC-NPCs and Schwann cells (SCs) was applied by Niapour et al. They wanted to take advantage of a cotransplantation strategy to overcome the low rate of neuronal differentiation of individual NPC transplantation [50]. Based on their results, significant motor function recovery was observed in all engrafted groups (NPCs, SCs, NPCs + SCs) when compared with the control group. Moreover, in comparison with the two individual transplantation groups, the greatest functional recovery was observed in the hESC-NPCs/SCs transplanted group with significantly increased expression of TUJ1 and MAP2, and decreased expression of GFAP at the fifth week after transplantation. Their study suggested that the cotransplantation of hESC-NPCs with SCs might be a feasible strategy to provide a sufficient synergistic effect to enhance neuronal differentiation and to simultaneously suppress glial differentiation, ultimately promoting functional recovery.

Besides glial cell activation, motor neuron loss is considered as another major characteristic of spinal cord injury which contributes to motor functional deficits, especially at the cervical level. According to the review from Nogradi et al., they suggested that the application of ESC/iPSC-derived motoneuron grafts is an efficient way to replace missing motoneurons which result from spinal cord injury [35]. The grafted motoneurons may be able to reinnervate the denervated muscles by extending their axons along the entire length of reimplanted ventral root and reach the muscles to restore limb locomotion function, rather than partially restoring integrity of the lesion site with local neuron or axon regeneration [117]. In vitro, a typical electrophysiological action potential of ESC-derived motor neuron can be elicited, and several physiologically active growth factors can be expressed and secreted by ESC-derived motor neuron progenitor (MNP). These include neurotrophin-3 (NT-3), neurotrophin-4 (NT-4), nerve growth factor (NGF), and vascular endothelial-derived growth factor (VEGF), can promote neurite branching and neuronal surviving [51]. *In vivo*, in order to test function and to observe the cellular behavior, Rossi et al. transplanted ESC-derived MNPs into the cervical lesion site of an adult rat model of SCI. When compared with vehicle control group, a series of significantly better results, that included enhanced sprouting of endogenous serotonergic (5-HT) projections, enhanced survival of endogenous neurons, enhanced gross tissue sparing, and decreased phosphorylation of stress-associated protein kinase which can result in apoptosis, immune activation, and inflammation were observed by them [51]. In addition to the recovery of motor function, the attenuation of tactile hypersensitivity and the recovery of general sensory function is another target which scientists want to achieve in research for a treatment for SCI. The upregulation of decreased GABAergic system activation after SCI was verified to have a role of relieving the pain-like response in rat hemisection SCI model [118]. For this reason, another kind of ESC/iPSC-derived neuron, ESC/iPSC-derived GABAergic neuron, was introduced into transplantation experiments for rescuing impaired sensory function post SCI by generating GABA around the lesion site during a long-term period [52]. Kim et al. evaluated the mechanical sensitivity of the hind paws by measuring paw withdrawal thresholds (PWTs) in the hemisection rat model on the application of a von Frey filament. After the intrathecal transplantation of ESC/iPSC-derived GABAergic neurons, a significant reversal of decreased PWTs was assessed beyond posttransplantation week 5 when compared with control group. Interestingly, the evoked response of wide dynamic range (WDR) neurons, which are responsive to all sensory modalities (thermal, chemical, and mechanical), to three different stimuli (brush, pressure, and pinch) in ESC/iPSC-derived GABAergic neuron transplanted group were significantly restored from a hypersensitive condition, to a level similar to the sham operation group. Importantly, all these phenomena could be blocked by the administration of GABA receptor inhibitors. Therefore, their study showed that a transplantation strategy using ESC/iPSC-derived GABA neurons may be a potential solution for the loss of sensory function after SCI.

Beyond the direct transplantation of derived neurons, transplantation of neural supporting cells remains attractive to scientists and ESC/iPSC-derived oligodendrocyte progenitors are one of the optimal options. In 2005, improved axon remyelination and motor function in a rat contusion SCI model by means of ESC-derived OPC transplantation was reported by Keirstead et al. [53]. Furthermore, ESC-derived OPCs demonstrated some distinctive immunological characteristics and were largely resistant to killing by human NK cells as well as to the lytic effect of antibodies [119]. With the capability to restore motor function via remyelination and specific immune-properties which suggest that these cells could be weakly immunogenic in nature and may not be rejected by the immune system, ESC-derived OPCs exhibit favorable properties for further development as a potential therapy for SCI. Kerr et al. derived OPCs from human ESCs and then injected these cells into a contusion SCI model in rats during the acute phase after injury. After eight days of transplantation, oligodendrocyte markers, including CNPase, GalC, Olig1, O4, and O1, were detected in the grafted ESC-derived OPCs. These grafted cells were reported to survive for a minimum of eight days and to migrate away from the injection sites to integrate into the injured spinal cord tissue. Some increased neurological responses were demonstrated in the transplanted group through behavioral and electrophysiological assessments compared to control groups [54]. In another study, Sharp et al. transplanted ESC-derived OPCs into a cervical contusion rat model to assess the restoration of forelimb motor function and to examine neural tissue protection from lesion pathogenesis. From the fourth week till the ninth week after transplantation, a significantly improved forelimb stride length was measured when compared with the control group. In the nontransplanted control group, a characteristic injury-induced cavity was observed with a lack of neural cells and axons in the border area surrounding the cavity. Moreover, perivascular cuffing and inflammatory infiltrates were identified in the lesion site which suggested dynamic, ongoing pathology. Meanwhile, in contrast to the control group, widespread white and gray matter sparing was observed in the lesion sites of the transplanted group, and most importantly, no injury-induced cavitation was identified. Furthermore, unlike the nontransplanted spinal cords, there was reduced demyelination and more oligodendrocyte remyelinated axons than schwann cell-remyelinated ones in the ESC-derived OPC transplanted spinal cords [55]. 

Although the transplantation of ESC/iPSC-derived neurons or OPCs has achieved promising results, combinatorial strategies have also been tested. Erceg et al. derived motoneuron progenitors (MPs) and OPCs from ESCs through different protocols, respectively, and then verified the efficacy of functional recovery promotion by MPs and OPCs, together, in a complete transection SCI rat model. As they expected, when compared with single-cell treatment and control group, the combined treatment group had significantly better BBB scores with significantly higher amplitude of motor-evoked potential (MEP) in electrophysiological evaluation at the end of experiment [56]. A similar positive result was reported in another combinatorial study by Salehi et al, who transplanted olfactory ensheathing cells (OECs) and ESC-derived motoneurons (MNs) into contused SCI rats [57]. This cotransplantation strategy rescued a significantly greater percentage of spared spinal cord tissue from contused lesion, and successfully enhanced remyelination after injury. The survival of grafted ESC-derived MNs in cotransplanted rats was sufficiently supported by OECs and, the number of surviving ESC-derived MNs in the cotransplanted group was significantly higher than in the single cell type transplantation with ESC-derived MNs group. A significant recovery of hindlimb function was observed in rats in the cotransplanted groups, together with improved histopathology.

## 5. Mesenchymal Stem Cells (MSCs)

Mesenchymal stem cell (MSCs) lineage is a kind of self-renewing and multipotent stem cell, which was initially identified from the bone marrow (BM) [120, 121]. In the adult human bone, the population of MSCs is rare, approximately 0.001%–0.01% of the total population of nucleated cells in the marrow [122]. However, human MSCs can be easily obtained from bone marrow by simple iliac crest puncture, and they are biologically safe and have been used extensively for transplantation in patients suffering from hematological cancer [23]. 

According to the statement of International Society for Cellular Therapy, the definition of multipotent MSCs must be fulfilled to a minimum criterion [123]. First, MSCs must be plastic-adherent when cultured in standard conditions. Second, MSCs must express CD105, CD73, and CD90, and lack the expression of CD45, CD34, CD14, or CD11b, CD79a, or CD19 and HLA-DR surface molecules. Third, MSCs must be able to differentiate to osteoblasts, adipocytes and chondroblasts *in vitro*. MSCs are able to be differentiated *in vitro* into osteoblasts, chondrocytes, adipocytes, neural cells, and even myoblasts [122, 124]. Within the field of regeneration research after CNS injury, MSCs are being advocated as a promising cell source for repair. The isolation of a population of multipotent stem-cells from human bone marrow [122], and demonstration of spontaneous neuronal differentiation of MSCs implanted into both irradiated mice [125, 126] and humans [127]; along with isolation of subtypes of nonhematopoietic MSCs capable of neuronal differentiation, have paved the way for their clinical use in neurorestorative approaches [124, 128, 129]. 

In stem cell therapy research for SCI, the application of MSCs is favored by some researchers because of the following excellent properties. First, the acceptance from the donor and the isolation from cryopreservation are relatively easy and simple [130, 131]. Second, the expansion of cells to clinical scales can be achieved in a relatively short period of time [132]. Third, the preservation of MSCs with minimal loss of potency can be performed conveniently [133]. Fourth, transplanted MSCs are capable of decreasing demyelination, reducing neural inhibitory molecules, of promoting axonal regeneration, and of guiding axon growth [134]. Lastly and importantly, there are no reports of adverse reactions to allogeneic versus autologous transplants, and allogeneic MSCs are well tolerated and do not elicit immediate or delayed hypersensitivity reactions [135, 136]. Carrade et al. injected equine allogeneic and autologous umbilical cord derived mesenchymal stem cells (UMSCs) twice into horses intradermally [135]. After the first injection, no adverse local and systemic responses within 7 days after injection were observed, except some minor wheal formations which were characterized as mild dermatitis and fully resolved by 48–72 hours. The second injection was 3-4 weeks later, and they reported no more significant physical and histomorphologic alterations compared with the first injection. This result indicated that neither the immediate, cytotoxic, immune-complex, and delayed hypersensitivity reactions, nor the graft-versus-host responses can be elicited by transplanted UMSCs. 

Azizi et al. (1998) reported spontaneous differentiation of human bone-marrow-derived stromal cells into astrocytes following implantation into the striate body of adult rats [137]. These cells, however, did not transform into neurons. Shortly after, Mezey et al. [126] and Brazelton et al. [125] simultaneously described spontaneous acquisition of cells bearing neuronal antigens, from bone marrow cells infused intraperitoneally in rats which had migrated to the brain of host animals. Mezey et al. used male-rodent mesenchymal cells, implanting them into females with congenital bone marrow aplasia. They confirmed neuronal differentiation through NeuN expression by immunohistochemical staining and confirmed cells as being those of the donor by using in situ hybridization of the Y chromosome, a difficult to execute technique yielding substantial unspecific punctiform staining patterns, potentially misinterpreted as Y chromosome. Notwithstanding, they reported that 0.3 to 1.8% (depending on age of recipient) of neuronal cells in the host rat forebrain were derived from the donor. In the second study, the authors employed transgenic rats whose cells constitutively expressed green fluorescent protein (GFP). Bone-marrow-derived stromal cells were extracted from these animals and subsequently implanted intravenously into irradiated rats with no viable bone marrow. They reported immunostaining for NeuN and high-molecular-weight neurofilament protein (NFH) coexpressing GFP in different cell types from olfactory bulbs of the host rats. 

Transplantation of MSCs in SCI animal models has been applied by several groups to promote sensorimotor function recovery and bladder function recovery via neural lineage differentiation, neurotrophic paracrine effects and posttrauma inflammation regulation (Table 2). As Nakajima et al. reported, the activation of macrophages in the post-SCI inflammatory environment can be regulated by the transplantation of MSCs [58]. After transplantation into the contusion epicenter, the undifferentiated MSCs significantly upregulated the level of IL-4 and IL-13, and downregulated the level of TNF-alpha and IL-6. These changes of inflammation factors resulted in the shifting of macrophage phenotype from M1 (iNOS- or CD16/32-positive) to M2 (arginase-1- or CD206-posistive). With the alteration of macrophage phenotype, more preserved axons, less scar tissue formation, and increased myelin sparing were observed, furthermore, locomotion recovery in the MSCs transplantation group was confirmed. In another MSCs transplantation trial, Karaoz et al. claimed significant motor recovery in the MSCs implanted group, however, only Nestin+/GFAP+ astrocytic-like cells were observed at 4 weeks after transplantation [59]. By implanting human MSCs into the contusion rat model, more rapid restoration of hindlimb function was achieved when compared with other control groups, but significant differences of BBB scores and coupling scores among all groups were not obtained. More importantly, bladder function was not restored in either group [60]. In addition to motor function deficits and bladder dysfunction, neuropathic pain is also a common and debilitating symptom in SCI patients which is induced by abnormal neuronal activities in the spared tissue surrounding the lesion site. In order to clarify the relationship between chronic inflammation and the therapeutic effects of MSCs on sensory deficits, Abrams et al. evaluated chronic inflammation, posttrauma cyst formation, and mechanical and thermal sensation thresholds of contusion SCI rats treated with MSCs transplantation [61]. After MSC injection at three different sites (the lesion site, rostral and caudal to the lesion), the injury-induced sensitivity to mechanical stimuli was significantly attenuated, although no effect was observed on injury-induced sensitivity to cold stimuli. More importantly, GFAP + reactive astrocytes and ED1+ macrophages/microglia, assessed as a measure of the chronic inflammatory response, were significantly attenuated by MSCs administration. The improvement of locomotor function in SCI rats by means of MSCs transplantation was also reported.

However, the therapeutic *in vivo* application of MSCs for spinal cord injury might face a series of challenges which include low survival rate of grafted cells (5–10%), the lack of neural differentiation, glial scar formation, cystic cavity formation, the inhibitory cellular environment, the transplantation time point, and the graft/host immune responses [58, 64–66]. In addition, different transplantation routes can also bring different outcomes after MSCs transplantation. In a comparison experiment, Kang et al. compared the BBB motor scores of SCI rats between intravenously (IV) and intralesionally (IL) transplanted groups [62]. The fates of engrafted allogenic MSCs in two different groups were also investigated. Based on their results, the NeuN positive neural differentiation and CC-1 positive oligodendroglial differentiation of engrafted MSCs was observed in the IL group, and GFAP positive astrocyte differentiation was observed in the IV group. Meanwhile, the expression of both BDNF and NGF in the IL group was significantly higher than the IV group. This phenomenon was suggested to be related to the absolute number of the engrafted MSCs. Regarding motor function recovery, both MSC transplantation groups achieved significantly better outcomes than the control group (BBB scale 6.5 ± 1.8). The BBB scores in the IV group (11.1 ± 2.1) was significantly better than the IL group (8.5 ± 2.8). The authors suggested that the nonfavorable motor function improvement in IL group might be related to the additional injury during the transplantation in the intralesional injections. By means of intravenous transplantation of LacZ reporter gene transduced MSCs in the earlier postinjury infusion time, Osaka et al. reported significantly improved locomotor recovery in severe contusive SCI rats, and they suggested that the minimal invasive, intravenous cell administration is a prospective therapeutic approach in acute and subacute SCI [63]. Mothe et al. investigated the effects of another transplantation approach, intrathecal transplantation, with neural stem/progenitor cells (NS/PCs) and bone-marrow-derived mesenchymal stromal cells (BMSCs) [64]. Most of transplanted cells were showed to remain in the intrathecal space, and neither NS/PCs nor BMSCs migrated into the parenchyma of the injury site.

After implantation into the injured spinal cord, the neuronal differentiation of MSCs *in vivo* is not efficient and the lack of neuronal markers expression has been reported in some transplantation studies [64–66]. Without neuronal differentiation, the engrafted MSCs may generate a favorable environment for functional recovery through modulating the post-SCI inflammatory response and by having neurotrophic paracrine activity [58, 64–66, 138]. As Boido et al. reported, significantly reduced lesion volume and improved hindlimb sensorimotor functions were observed after mouse MSCs were transplanted into the lesion cavity of compression SCI mouse model, even though the engrafted MSCs were observed to be neuronally undifferentiated and astroglial and microglial activation was not altered [65]. Gu et al. also reported similar results, the reduced volume of post-SCI cavity and increased spared white matter were observed after transplantation of bone marrow mesenchymal stem cells into the epicenter of the injured spinal cord of rats [66]. Interestingly, despite the lack of expression of neuron, astrocyte, and oligodendrocyte cell markers, an increase in the number of axons in MSCs transplanted rats was confirmed via transmission electron microscopic examination. In the *in vitro* experiment of the same study, Gu et al. investigated the paracrine activity of MSCs by means of a MSCs and spinal neuron coculture system. Their results confirmed the expression of brain-derived neurotrophic factor (BDNF) and glia cell line-derived neurotrophic factor (GDNF). 

The therapeutic effects of MSC transplantation on the sensorimotor deficits in animal SCI models have been clearly confirmed by a large number of studies [61, 63, 65, 67]. 

In order to overcome the potential problems associated with direct transplantation of undifferentiated MSCs, researchers have tested several modifications of transplantation strategies, such as pretransplantation neural differentiation, neurotrophic gene transduction, glial cell co-transplantation, and tissue engineering [67–75, 139–142]. The neural pretransplantation differentiation is the most commonly used strategy to promote the therapeutic effects of engrafted MSCs. Rodent MSCs are able to efficiently differentiate into neural precursors by culturing with basic fibroblast growth factor (bFGF), epidermal growth factor (EGF), and heparin [143]. One method of human MSC neural differentiation was described by Alexanian et al. in 2011 [67]. According to his method, human MSCs were exposed to histone deacetylases inhibitor (Trichostatin), DNA methyltransferase inhibitor (RG-108), biologically active form of cAMP, and phosphodiesterases inhibitor (Rolipram) in a medium consisting of NeuroCult/N2 supplemented with bFGF for two weeks before transplantation. Park et al., reported a new method to generate functional motor neuron (MN)-like cells from genetically engineered human MSCs [141]. They transduced motor neuron-associated transcription factor gene expression into the human MSC, then they treated the genetically engineered MSCs expressing Olig2 and Hb9 with optimal MN induction medium. By using an* ex vivo* model of SCI, they showed that these reprogrammed MSCs exhibited characteristics of MN-like lineage and are potentially therapeutic for autologous cell replacements.

Alexanian et al. injected neural modified bone-marrow-derived MSCs rostral and caudal to the T-8 lesion immediately after injury [67]. 12 weeks after SCI, locomotor function was significantly improved by the neurally modified MSCs, and the volume of lesion cavity and white matter loss were significantly reduced. However, the improvement of thermal sensitivity was not observed. Cho et al. transplanted neurally differentiated rat MSCs (NMSCs) into the epicenter of a contusive lesion, thereafter, the BBB scores, somatosensory evoked potentials (SSEPs) and motor evoked potentials (MEPs) were evaluated. Nine weeks after NMSCs transplantation, the recovery of motor function was reported, and significantly shortened initial latency, N1 latency and P1 latency of the SSEPs were observed [69]. Pedram et al. utilized a Fogarty embolectomy catheter to create a contusion lesion at T8-9 level of rats' spinal cord, then the autologous neural differentiated and undifferentiated MSCs were cotransplanted into the center of lesion cavity [70]. Five weeks after transplantation, the BBB scores in both cotransplantation group and predifferentiation group were reported to be significantly higher, when compared with undifferentiated group, respectively. However, no significant difference between cotransplantation and predifferentiation groups was observed.

In addition to neural predifferentiation, neurotrophic gene transfection has also been tested in some MSC *in vivo* studies. Liu et al. implanted bFGF transgene expressing rat MSCs into the SCI rat model and reported a significantly higher BBB score in the bFGF group when compared with control groups at 3 weeks after the injection. Furthermore, significantly more bFGF-positive neurons were observed in the bFGF group, and significantly higher optical density values of NF200-positive neurons and MBP-positive axons were also demonstrated in the bFGF group. Therefore, they suggested that the bFGF gene-modified MSCs might be effective in promoting axon regeneration and functional recovery after SCI [71]. In another *in vivo* study using gene modified MSCs, Zhang et al. investigated the therapeutic effects of Neurotrophin-3 (NT-3) gene modified MSCs in an ethidium bromide (EB)-induced demyelination SCI model of rats [72]. 21 days after the administration of NT-3 modified MSCs, locomotor function was improved, and similar to that in the saline injured control group. The improvement was significantly better than the other groups which include MSC group, LacZ gene modified group, and EB injured group. Similar improvements of spinal cord evoked potentials (SCEP) amplitude and SCEP latency were also achieved in the NT-3 modified MSCs group. Via immunostaining, significantly higher number of NG2- and APC-positive engrafted MSCs were observed in the demyelination site of the spinal cord after transplantation of NT-3 modified MSCs at the end of experiment.

In order to provide a favorable environment for neural regeneration and to support the survival of implanted cells and their neural differentiation, the use of biologic scaffolds has drawn increasing interest. Zurita et al. developed a biologic scaffolds system from blood plasma, called platelet-rich plasma (PRP) scaffolds. According to their report, most of the cocultured human MSCs demonstrated optimized capabilities of survivalg and neural differentiation after the administration of BDNF [142]. In 2011, a gelatin sponge (GS) scaffold system, which was constructed by ensheathing GS with a thin film of poly-(lactide-co-glycolide) (PLGA), was reported by Zeng et al. Based on their work, this GS scaffolds system was able to provide a favorable environment for seeded rat MSCs to adhere, to survive, and also to proliferate. After they transplanted GS scaffolds seeded with rat MSCs into the rat SCI model, a promising result which includes attenuated inflammation, promoted angiogenesis, and reduced cavity formation was reported [73]. In 2012, a combinatorial strategy using a similar PLGA scaffolds system and human MSCs was employed by Kang et al. to evaluate the therapeutic effects on motor function improvements. After PLGA scaffolds seeded with human MSCs were transplanted into a completely transected SCI rat model, significantly higher BBB scores were demonstrated. More importantly, the amplitude of motor-evoked potentials (MEPs) in the combinatorial strategy treated group was significantly higher than the other control groups. In addition, implanted cell survival, neural differentiation, and axon regeneration in the combinatorial strategy group were confirmed by immunohistochemical staining images [74]. In another study, a combination of Matrigel and neural-induced adipose-derived MSCs (NMSCs) was applied by Park et al. to investigate the therapeutic effects on functional recovery from SCI in dogs. 8 weeks after the administration of the combination of Matrigel and NMSCs, a significantly better functional recovery was observed as higher BBB and Tarlov scores. Meanwhile, the reduced fibrosis from secondary injury processes, decreased expression of inflammatory and astrogliosis markers, increased expression of neuronal and neurotrophic markers were also confirmed [75].

Although the bone marrow is the main source of MSCs, scientists have been seeking other sources because bone-marrow-derived cells are highly vulnerable to viral infection and the significantly increased cell apoptosis and the loss of differentiation capability that occurs in these cells with age [144]. Alternative sources of MSCs have been identified by researchers, such as, adipose tissue [140], amniotic fluid [145], placenta [145, 146], umbilical cord blood (UCB) [138, 147], and in several fetal tissues including liver, lung, and spleen [148]. Among all the substitutes for BM-derived MSCs, the UCB is the best choice with many advantages of UCB as compared to BM. The collection of cord blood units is more easier and noninvasive for the donor, the UCB units can be stored in advance and are rapidly available when needed, and the MSCs from UCB is more primitive than the MSCs collected from other sources [149, 150]. Importantly, they are less likely to induce graft-versus-host reactivity due to their immaturity [151]. Ryu et al. investigated the effects of MSCs from different tissues on the regeneration of injured canine spinal cord, which are fat tissue, bone marrow, Wharton's jelly and umbilical cord blood [152]. Although the differences among four experimental groups were not detected in this study, more neural regeneration and anti-inflammatory activity were observed in the experimental group with umbilical cord blood derived MSCs.

Guo et al. [76] induced human umbilical cord mesenchymal stem cells (hUMSCs) into Schwann-like cells *in vitro* and grafted these cells into the lesion site of SCI rats. A partial recovery of motor function was reported. Furthermore, neurotrophin-3 (NT-3) administration combined with *in vivo* transplantation, significantly increased the survival of grafted cells and improved the behavioral test results compared to the cell transplantation only group. Meanwhile, Shang et al. [77] transplanted genetically modified NT-3-hUMSCs to the spinal cord injured rats, and the Basso, Beattie and Bresnahan (BBB) scores and grid tests were applied to evaluate the functional recovery at the end of 12 weeks after SCI. In addition to the promotion of transplanted cell survival, significantly better motor function recovery compared to hUMSCs group was achieved in the NT-3-hUMSCs group. This was associated with intensified 5-HT fiber sprouting, more spared myelin, and reduced cystic cavitation. 

The pathological processes at the lesion site in SCI evolve over time, from acute phase, subacute to chronic phase, therefore transplantation at different times postlesion, may have varied effects. The comparison of three different transplantation times (12 hr, 1 week, and 2 weeks after injury) has been explored by Park et al., they injected 1 × 10^6^ canine UMSCs into the balloon-induced compression lesion site of experimental dogs in different time groups [153]. The significant improvement of Olby and Tarlov scores, which were used to evaluate functional recovery of the hind limbs, was observed in the 1 week transplantation group, and the accompanying increase in the expression of neuronal markers and decreased expression of inflammation markers were measured as well. In addition, less fibrosis was demonstrated in the 1 week group compared to other groups. Therefore, it is reasonable to conclude that one week after SCI may be the best time point for the further development of therapeutic studies to obtain neuronal regeneration, reduced fibrosis, and eventual function improvement.

In most studies, assessing the long-term effects of treatments is technically difficult due to associated risks of weight loss, urinary infection, and sepsis in injured animals. However, a 3 year long-term effects study of hUMSC transplantation in dogs with SCI was reported by Lee et al. in 2011 [78]. The hUMSCs were transplanted into the balloon injured lesion site in seven experimental dogs. Despite two transplanted dogs dying within one month after transplantation, four of the five surviving experimental dogs survived for three years. These four dogs had restored the hind-limb motor functions (BBB scores) with significant improvement at three years after injury and deep pain recovery was detected from 5 days post injury. Immunohistochemical staining revealed remyelination with many myelin protein-zero positive axons which is the major structural protein of peripheral myelin.

## 6. Neural Stem/Progenitor Cells

Neural stem/progenitor cells (NS/PCs) were first demonstrated in the subventricular zone of the mouse in 1989 [154] and were isolated from the mouse striatal tissue and subventricular zone for the first time in 1992 [7, 155]. These cells were capable of self-renewal and generating the main phenotypes (neurons, astrocytes, and oligodendrocytes) of CNS cells *in vitro* and* in vivo* [156]. After transplantation into the injured spinal cord, NS/PCs generate mature neural phenotypes and provide neural functional recovery in some SCI models [156].

*In vitro* culture, NS/PCs can be maintained in a particular and unique living cluster shape to proliferate called a neurosphere. This neurosphere culture system is the main method of neural stem/progenitor cells' study, which was developed by Reynolds and Weiss [155]. Neurospheres are mainly composed of two sorts of cells, one which is an electron-dense and slowly dividing neural stem cell population and their progeny which are immunopositive to actin, weakly positive to vimentin, and nestin-negative, and another population of electron-lucent and fast-dividing progenitor cells which are actin, vimentin and nestin positive [155, 157]. Epidermal growth factor (EGF) and fibroblast growth factor (FGF) are two vital nutritional growth factors that can promote neural progenitor and stem cell growth *in vitro* and *in vivo* [158, 159]. The EGF and FGF receptors are widely expressed in the cytoplasm and nucleus of neural stem/progenitor cells. The amount of sphere component nestin-positive progenitors determines the neurosphere size, and changes in the different cellular populations within neurospheres can result in the alterations of the survival, proliferation, and differentiation capabilities of their neural stem/progenitor cells [160]. As Weible and Chan-Ling reported [161], with the presence of bone morphogenetic protein 4 (BMP4) and leukemia inhibitory factor (LIF) in medium, the portion of oligodendrocytes and neurons can be significantly decreased to 3% and 16%, respectively, and the portion of GFAP+ neural precursor cells is increased to 79%. Based on this study, the neurospheres culture system is able to provide a pure population of astrocytes, which have been extensively utilized for stem cell research. However, the neurospheres culture system cannot be used as a precise assay for assessing clonality, number and fate of stem cells due to the intrinsic dynamic property of neurospheres [162]. Thus, on the basis of proliferative potentials, Louis et al. developed a novel assay for neural stem cells research, the Neural Colony-Forming Cell Assay, which is capable of discriminating NSCs from various progenitor cells, and more accurate regulating of NSCs for specific applications in further experiments or therapeutic use [163].

The *in vivo* transplantation of neural stem/progenitor cells has been widely applied in the therapeutic study of SCI. Scientists have attempted to restore neural functions via a number of different strategies including neuronal differentiation, axon regeneration, remyelination, and nutrient secretion (Table 3). The survival rate and cellular character alternations in a long period are vital to the transplantation therapy. The long-term properties of human spinal cord-derived neurospheres were examined by Åkesson et al. [79], they were successful in culturing neurospheres *in vitro* with EGF, bFGF, and CNTF for up to 25 passages for about 350 days. After 18 passages expansion *in vitro*, the differentiated neurons and neural cells were transplanted into the spinal cord lesion of rats. The minimum survival time for the majority of transplanted cells was 6 weeks, and the expression of neuronal and astrocytic phenotypic markers were observed in these surviving cells. Their results suggested that the neurospheres can be well maintained, expanded, and remain multipotent for a long period of time *in vitro*. The results demonstrated that these long-term cultured cells still have a promising survival rate and differentiate in the injured spinal cord *in vivo*, although most of them likely differentiate into astrocytes. 

In most cases, *in vivo* transplanted NSCs have shown a preferential capability of differentiating into glial lineages, especially astrocytes [164]. The direct transplantation of NSCs or NPCs are not always efficient for functional recovery after SCI. Webber et al. [80] transplanted fetal NPCs, derived from fetal rats, into the dorsal column lesion site of adult rats. Although most of the grafted cells survived and remained around the lesion, only minor sensory function improvement was observed, and the motor function recovery was not restored. This result was probably a result of the high differentiation rate (40%) of grafted stem cells into glial cells, low neuronal differentiation, and the failure of axon regeneration beyond the lesion site. Tarasenko et al. treated hNSCs with bFGF, heparin, and laminin for priming before transplantation. Then, they transplanted these primed hNSCs into the contusion lesion of rats at the same day or 3 or 9 days postinjury. Compared with the unprimed group, the best results with optimized survival rate, neuronal and oligodendroglia differentiation, and improved trunk stability were obtained 3 months after the engraftment in the primed and 9 days postinjury transplantation group [81]. They claimed that human neural stem cell fate determination *in vivo* might be influenced by the predifferentiation treatment prior to grafting, and furthermore the functional improvement is related with the transplantation time point after injury, and the newly differentiated neurons and oligodendrocytes. Yan et al. [82] reported that the spinal cord microenvironment can probably change the differentiating fate of grafted NSCs. The centrally located NSCs appeared to differentiate into neurons, and the other cells located under the pia membrane tend to have an astrocytic phenotype. Moreover, the lesion microenvironment in the white matter of the spinal cord can markedly promote the differentiation of NSCs into astrocytes.

Grafted NSCs can also differentiate into neurons with certain pretreatments. Remyelination, and synaptic contact reformation is essential for the restoration of spinal cord circuitry which are the structural and physiological elements for functional recovery. Yasuda et al. transplanted shi-NS/PCs, which were obtained from myelin-deficient shiverer mutant mice, into the lesion site of rats in order to compare the capability of remyelination with wt-NS/PCs. At the end of experiment, they claimed that the remyelination capability of wt-NS/PCs was vital to motor and electrophysiological functional recovery [83]. Hwang et al. transplanted Olig2-NSCs, which were transfected by retrovirus with Olig2 transcription factor expression, into contused spinal cord [84]. They observed high proliferative activity of Olig2-NSCs in the experimental group by 7 weeks after transplantation, and the increased volume of spared white matter and reduced cavity volume were observed as well. Further, thickened myelin sheath was detected, which may have been induced by the differentiation of NSCs into oligodendrocytes. More importantly, significant locomotor recovery of the hindlimbs was also measured. Alexanian et al. isolated A2B5(+) NG2(+) NPCs from hNPC neurospheres, and then transplanted them into SCI rats. As a result, compared with NCAM(+) A2B5(+) group and NCAM(+) A2B5(+) group, the significantly improved locomotor and sensory functional recovery was obtained in the A2B5 (+) NG2 (+) group [85]. Both of the studies above indicated that oligodendrocyte differentiation from grafted neural stem/progenitor cells is vital to the functional recovery promoted by remyelination by oligodendrocytes in the CNS. Besides remyelination, synaptic contact reformation is also important for the reconstruction of neurofunctional circuitry. Yan et al. [82] grafted NSCs from human fetal spinal cord into the lumbar cord of adult nude rats. The large-scale differentiation into neurons, axon regeneration, and extensive synaptic contacts reformation with host motor neurons was observed. As they reported, the newly differentiated neurons integrated into the host neural circuits, which indicated the possibility of neural circuitry restoration in the traumatically injured spinal cord. In addition to the transplantation of single cell types, the combined dual-type or multitype strategies are also being actively pursued. Wang et al. [86] injected NSCs and olfactory ensheathing cells (OECs) into the spinal cord lesion of rats at 7 days post-SCI, and reported hindlimb locomotor functional recovery at twelve weeks post transplantation. Novel NF200 positive fibers which crossed through the injured region were observed by them, however, they did not examine the axon remyelination and synapse formation. The optimal time point for cellular transplantation after spinal cord trauma has not been established till now. The most common transplantation time window ranges from 7 d.p.i. to 10 d.p.i. However, the largest population of SCI patients is composed of chronically injured individuals. The inflammatory response, glial cell activation, and the inhibitory microenvironment that exists in the acute phase after trauma largely acts as a negative obstruction to any form of cellular therapy. On the other hand, the pathological alterations of the lesion site in a chronic patient may not be reversible due to the formation of a glial scar, the permanent demyelination/dysmyelination of spared axons, and the apoptosis of spared neurons. Therefore, the optimal transplantation time-window most probably lies in the subacute phase. The study of Salazar et al. demonstrated significantly improved locomotor recovery in an early chronic spinal cord injury mouse model after NSC transplantation [87]. More importantly, they showed that most of the transplanted NSCs had differentiated into oligodendrocytes and neurons and that astrocytic differentiation was rare. The authors also reported the integration of transplanted human NSCs with host cells. 

## 7. Olfactory Ensheathing Cells

Olfactory Ensheathing Cells (OECs) are considered as a special class of glial cells which exist in both the PNS and CNS, and share certain features and functions with astrocytes as well as Schwann cells [165]. OECs are present in the olfactory epithelium, where neurogenesis occurs throughout adulthood. The olfactory epithelium (OE) is composed of two kinds of neural stem cells, which are the globose basal cells (GBCs) and the horizontal basal cells (HBCs). GBCs are the main resource for homeostatic neurogenesis that leads to the birth of neurons and other cellular populations such as OECs. Unlike the GBCs, HBCs are normally quiescent, but they can be activated to generate novel GBCs to reconstruct the cellular populations of OE after injury [166, 167]. OECs were identified as an elongated shape with thin laminar processes that ensheath olfactory nerves *in situ*, but the morphologies of cultured OECs are distinct, from flat shape to bipolar and tripolar, moreover, there are also various antigenic differences. These heterogeneities may be caused by the different origins of the olfactory tissue used, the age of donor, the method of isolation, and culture conditions, and can also be affected by extracellular and intracellular molecules [168]. This kind of property is thought to allow OECs to transform themselves within different morphological and antigenic types to exhibit different functions and to adapt various environments [168]. When OECs act as Schwann cells with the same bipolar appearance, they can produce similar axon growth molecules, although the remyelination ability is poorer than Schwann cells [169, 170]. When they are transformed to astrocyte-like flattened cell shapes, a GFAP positive cellular supporting structure can be detected [165]. Nevertheless, compared with Schwann cells, OECs are more likely to rescue neural function in the injured spinal cord by virtue of their cell-specific properties. The bridging effect of transplanted OECs on regenerated axons of from dissected dorsal root into spinal cord was reported by Li et al. [171]. Importantly, OECs were shown to be able to repress astrocyte proliferation and reactivity *in vitro*, activated astrocytes after injury are the main source of the glial scar [172].

On account of their neuronal regeneration-promoting potential and their ability to support axonal outgrowth, OECs have been tested in *in vitro* and *in vivo* experiments for their regeneration promoting effects in SCI [173, 174] (Table 4). Although *in vivo* functional recovery by means of OEC transplantation has been reported by several groups, the mechanism of the regeneration-promoting ability is still far from clear. A recent study reported electrophysiological evidence of the recovery of motor-evoked potentials and axonal regeneration after OEC injection into a complete transection lesion [88]. But other groups have shed doubt on the functional improvements induced by OECs grafts, and have suggested that they are caused by a trophic support mechanism and not the birth of new neurons, which means that the therapeutic potential of OECs after SCI may be limited [89, 90]. Lu et al. reported that no significant axon growth promoting effect was detected in the OECs transplanted group, and no bridge-crossing phenomenon of corticospinal axons was observed beyond a dorsal column lesion [89]. Collazos-Castro et al. transplanted OECs into cervical contusion injury model of rats, neither dorsal corticospinal tract axon regeneration nor locomotor deficits recovery was demonstrated [90]. Furthermore, the result from the olfactory tissue transplantation study of Centenaro et al. [91] and Aoki et al. [92] also suggest that OECs may be of limited use in promoting recovery after SCI. They transplanted tissue pieces of olfactory lamina propria (OLP) and respiratory lamina propria (RLP) into the transection lesion site of adult rats. After grafting, similar hindlimb motor improvement, comparable spinal cord tissue sparing and sprouting in the lesion site was observed between the OLP and RLP groups. In addition, only limited supraspinal axonal regeneration was shown by retrograde tracing, even though a large number of 5-HT positive fibers were found next to the grafts. Therefore, they suggested that the limited functional recovery and neural reparative effects may not be exclusively related to OECs [91]. Aoki et al. transplanted the whole-layer olfactory mucosa into the completed injured rats, and only observed limited functional recovery [92]. 

All these negative results above may be attributable to a number of factors, such as the nature of cell donor, the tissue source, the injury models, graft cells preparation, the time point of transplantation, and transplantation procedures. Compared with olfactory bulb-derived OECs (OB-OECs), Richter et al. reported reduced cavity formation, better axon regeneration, and remyelination after transplantation of lamina propria-derived OECs (LP-OECs) [93]. Zhang et al. reported that LP-OECs can indirectly promote tissue repair, axonal regeneration and remyelination, and shrink the cavity after scar ablation and lamina propria tissue transplantation. However, motor function recovery was not achieved [94, 95]. The result from Yamamoto indicated that olfactory mucosal cells were not able to promote CST axon regeneration, despite restoration of fore-paw motor function [96]. 

Concerning the “transplantation Time-window point”, the inflammatory reaction and acute cellular response in the acute phase after injury is certainly antagonistic to neuronal regeneration, axonal extension, and grafted cell survivals surviving. *In vitro*, the apoptosis rate of OECs with the appearance of acute explants of spinal cord was demonstrated significantly higher than the chronic group [175]. The chronic lesion site was divided into three different histological zones from outside to center by Zhang et al. (1) Fibrotic zone, which consists of invading connective tissue. (2) Cellular zone, which is composed of invading Schwann cells. These Schwann cells might presumably migrate from the lateral dorsal roots. (3) Axonal zone, which is composed of spared and regenerated axons. After ablation of scars, the OECs from LP grafts increased the size of the cellular and axonal zones, more importantly, the absence of scar formation, the integration of repaired tissue with spared tissue, and remyelinated axons in the axonal zone were observed [94]. Muñoz-Quiles et al. [97] compared the motor function recovery after OB-OEC transplantation into completed transection injured rats among subacute (1 month after injury), chronic (4 month after injury), and nontreatment groups. At the seventh month after transplantation, all the treated rats had improved hindlimb motor function, and the improvement was significant when compared with nontreated rats. In addition, the final plateau of percentage of recovery in subacute transplantation group was reported 10% higher than the chronic group. Interestingly, they also showed the demonstration of regeneration of motor axons growing beyond into the lesion site, which indicates the lesion site-crossing phenomenon from rostral to caudal [97]. Based on these data therefore, we propose that the subacute or chronic cellular transplantation to bypass the acute phase after spinal trauma combined with scar ablation may be a potentially effective strategy. Considering the potential of secondary damage to clinical patients caused by scar ablation, cellular therapy during the subacute phase which occurs prior to the formation of the permanent glial scar may be more valuable and feasible for further clinical application.

After transplantation into injured spinal cord, the fate of grafted OECs can also be influenced by *in vitro* culture conditions. The survival of transplanted OECs and their properties of neuroprotection, neurotrophic factor expression, axon growth-promotion, and remyelination can be affected by the duration of pretransplantation culture and the purification methods used [98]. According to the report of Novikova et al, compared with the shorter preculture time (3 weeks), OECs with longer preculture time (7 weeks) are significantly less effective in protecting neurons and promoting axonal regeneration due to aging of the cells [98]. They also suggest that the differential cellular signal responses to disparate microenvironments within different purification methods used for preparation might induce distinct cellular behaviors after OEC transplantation.

Although several questions regarding the application of OECs have been raised, several recent studies support a protective/regenerative role [88, 102, 103, 176]. Electrophysiologically, OECs were confirmed to be able to preserve the function of circuitry with the evoked cord dorsum potentials and sensorimotor cortex potentials in the region of dorsal column lesion after transplantation [99]. In another study, Liu et al. comprehensively analyzed behavioral improvements, somatosensory and motor evoked potentials in rats after OECs transplantation. Significantly improved results were measured in OEC-treated rats compared with control groups despite the absence of retrograde labeling [100]. Furthermore, autonomic dysreflexia which can cause abnormalities in blood pressure, heart rate, and respiration in high level spinal cord injury was assessed in the study from Kalinčík et al. They reported the normalization of enlarged sympathetic preganglionic neurons by means of OEC transplantation in a transection SCI model [101]. This cellular morphological normalization was suggested to be meaningful for the recovery from autonomic dysreflexia although no effect on cardiovascular parameters was confirmed in the OEC-grafted group except a 25% shorter recovery time from hypertension [101]. Regarding motor function recovery, Tharion et al. assessed motor-evoked potentials and scores on the BBB scale after OECs transplantation. They reported significant improvement in the OEC-grafted group when compared with the control group. Furthermore, they reported one animal that was followed-up for 264 days after transplantation with the highest BBB score of 17 [102]. Improvements in respiratory function are vital for recovery post-high-level spinal trauma. By using a cervical contusion rat model, Stamegna et al. induced a persistent hemidiaphragmatic paralysis for assessing the therapeutic efficiency of OEC transplantation at 2 weeks postcontusion. At 3 months after transplantation, significant improvement of breathing movements, activities of the ipsilateral diaphragm and axonal sprouting in the lesion site was observed, suggesting that respiratory function was partially restored [103]. 

Although the application of only OECs has shown promise in the promotion of recovery after SCI, combinatorial approaches have also been utilized in order to boost efficacy. cAMP treatment [104], Neurotrophin-3 (NT-3) production via genetic modification [105], Laserponcture [177], and cotransplantation with other cells [57, 106, 178] have been combined with OEC transplantation. The weak intrinsic neuronal growth response has been shown to contribute to the failure of neuronal regeneration after SCI. The cAMP pathway has been shown to be critical for increasing this intrinsic capacity in neurons [179]. Bretzner et al. transplanted lamina propria-derived OECs into dorsolateral funiculus crush lesion site with cAMP infusion treatments. The authors reported a significant decrease of GFAP expression and cavity formation, with remarkable axon regeneration and both sensory and motor function improvement. Their study indicates the feasibility and efficacy of a combined strategy of OEC transplantation and intrinsic cellular signal enhancement to promote recovery after SCI [104]. As a member of the neurotrophic superfamily, NT-3 can counteract pathological factors post-SCI and promote the survival of neurons after SCI [180]. NT-3 can also stimulate neuronal regeneration and neurite outgrowth [181]. Ma et al. transplanted NT-3 gene-modified OECs, which can express NT-3 efficiently, into the contusion lesion of rats in order to promote better morphological and functional recovery when compared with simple OEC transplantation [105]. Based on their results, both axonal regeneration, which was verified via HRP retrograde tracing, and motor function recovery, which was assessed by BBB scoring, were significantly better in the combination group compared to normal OECs and the control group. 

In the development of therapeutic research for neural function recovery after SCI, distinct cellular functions specific to different transplanted cell lines have been identified in repairing damaged neural tissue. Theoretically, the combined application of different cell types may provide more benefits than single-celltype transplantation by means of synergistic effects. Salehi et al. reported significantly better recovery of rat hindlimb motor function, which was accompanied by significantly greater percentage of spared tissue, axon regeneration, and remyelination in the cotransplantation group of OECs and embryonic stem cell-derived motor neurons, when compared with the other single-celltype groups [57]. However, not all types of cells in combination confer a therapeutic advantage. Amemori et al. tried to develop a cotransplantation strategy which included OECs and MSCs, and expected a significant synergistic effect in neural function improvement. Unexpectedly, no significant differences of BBB scores and plantar tests were assessed among all groups at the end of the experiment, although some improvements of motor function were observed within different time points [106].

## 8. Schwann Cells

In the SCI patients and large animal models of SCI, a cystic cavity usually forms after injury, and a glial scar formed wall separates this cavity from the surrounding spared rim of white matter. At the edge of the glial scar, the regenerated axons regularly terminate in dystrophic endings, which means the termination of axon regrowth [182]. In response to overcome this serious obstacle of regeneration, developing an efficient corresponding bridging countermeasure becomes more and more urgent. After the spinal cord injury, the injured neurons can demonstrate an intrinsic capability of growth cone formation and axon extension initially, but all these “regenerating behaviors” are soon suppressed by the inhibitory microenvironment. When transplanted these injured neurons from the spinal cord lesion site are transplanted into a peripheral neural environment, they can completely recover as normal neurons electrophysiologically and morphologically [183]. The Grafted peripheral nerve segments in the spinal cord were reported to be capable of improving the recovery of behavioral and electrophysiological function *in vivo*, via axons regeneration, reformation of functional synapses with host neurons, neurotrophic molecule secretion, and by providing a permissive PNS-like environment providing [184, 185] (Table 5). Schwann cells (SCs), the myelinating cells of the PNS, play important roles in postinjury nerve regeneration by contributing to the axon regeneration and remyelination and forming guidance bands, bands of Büngner, for regenerating axons [186]. After transplantation into a demyelinated spinal cord slice *ex vivo*, SCs can stimulate the survival and intrinsic regeneration ability of damaged neurons by producing a number of neurotrophic factors which include NGF, BDNF, and CNTF [186]. In addition, the grafted SCs can also generate a variety of cell adhesion molecules and extracellular matrix proteins to support axonal growth as well, such as integrins, N-cadherin, N-CAM, L1, contactin, laminin, and collagens [187, 188]. More importantly, in order to achieve the goal of neural functional recovery by means of SCs treatment, the remyelination of demyelinated axons or newly sprouted axons must occur, as has been observed and confirmed [107, 189]. 

Traditionally, SCs were commonly isolated from peripheral nerves, and proliferated in culture to generate a large number of cells. Recently, the SCs used in SCI research have been derived from several categories of stem cells or neural progenitors directly, including mesenchymal stem cells [186], adipose-derived stem cells [190], and skin-derived precursors [191]. In an *ex vivo* experiment [186], Park et al. examined the neurotrophic effects of MSC-derived SCs with Neuro2A cells in a lysolecithin-induced demyelinated organotypic coculture system. The significantly enhanced axonal outgrowth of Neuro2A cells was promoted by the two specific neurotrophic factors: hepatocyte growth factor (HGF) and vascular endothelial growth factor (VEGF), which were secreted by MSC-derived SCs. Concurrently, a dramatic decrease in lysolecithin-mediated cell death was confirmed via the assessment of average number of TUNEL-positive cells per slice, which was decreased by 30% and 50% when compared with the MSCs treated vehicle group and control group, respectively. Xu et al. generated neurospheres from adipose tissue collected from embryonic mesoderm, and then successfully differentiated into SCs [190]. They cultured SH-SY5Y cells in the conditioned medium (CM) collected from SCs for 3 days in order to evaluate the effects of soluble factors secreted from SC-like cells. Compared with the results of the control group, beta-tubulin III positive neurite outgrowth was detected in around 31% of SH-SY5Y cells in the CM treated group. After 14 days, the multilayer membranes composed of myelin structures were observed surrounding the PC12 cell neurites cocultured with SCs via electron microscopy. Biernaskie et al. developed an efficient protocol for generating Schwann cells from skin-derived precursors (SKPs) which can be isolated from the dermis of both rodent and human skin [191]. They transplanted SKP-derived SCs or SKPs into a murine contused model to examine their repair promoting abilities in the injured spinal cord [107]. Although both the SKPs and SKP-derived SCs contributed to the reduced size of contusion cavity and remyelinated axons in the lesion site at 12 weeks after transplantation, SKP-derived SCs provided more promising results when compared with the SKPs transplantation group, including lesion site bridging effect, increased size of spared tissue, and reduced reactive gliosis [107]. Functionally, a significant enhancement of locomotor recovery was achieved in the SKP-derived SCs transplantation group, although there was no restoration of sensory function. Agudo et al. assessed the therapeutic potential of Schwann cell precursors (SCP) in an acute SCI model by immediate cell injection into the lesion site after surgery [108]. Unlike the SCs, they reported that SCPs started to proliferate rapidly right after the transplantation to fill the site of cavity where an injury-induced cavity is present in the control group. Within the cystic cavity, SCPs induced angiogenesis which was verified by the appearance of typical immunostained blood vessels with the expression of smooth muscle actin (SMA). Instead of the proliferation, which had been reduced to less than 40% after 4 weeks after transplantation, the maturation of SCPs into S100b positive SCs was observed. 8 weeks after transplantation, the SCP-differentiated SCs group had significantly reduced glial scar formation with significant reduced expression of GFAP. More importantly, the grafted cells successfully integrated into the host tissue, and a robust bridging effect was observed extending rostrocaudally. The regenerated BDA-labeled CST axons, which successfully crossed the lesion site, were assessed using anterograde tracing. These regenerated axons were also confirmed to be remyelinated by P0 positive myelin. However, motor function was not significantly different between the SCP and vehicle group.

In order to overcome the limitations of one particular cell type and to maximally tap into the potential of SCs; the genetic modification, combined treatments, and cotransplantation have all been used in SCI research. Glial cell line-derived neurotrophic factor (GDNF) promotes the survival of dopaminergic and motorneurons and increases axonal regeneration [192]. Deng et al. examined the interaction between genetically modified GDNF overexpressing SCs (GDNF-SCs) and reactive astrocytes in a specific guidance channel culture system *in vitro *[193]. According to the results of their study, GDNF-SCs suppressed the expression of GFAP and CSPG of reactive astrocytes, and then induced robust migration of astrocytes into the GDNF-SC transplants with elongated processes which extended in parallel to the regenerated axons. Importantly these axons were remyelinated (SMI-31 positive) by GDNF-SCs. Overall, their work indicates a novel and attractive strategy to control the reactive astrocyte induced inhibitory environment and to promote greater axonal regeneration, remyelination, and functional recovery following SCI. 

In addition to genetic modifications, combining SCs with different kinds of matrices and scaffolds have also been attempted in recent years [109, 110]. Pearse et al. suggested that the traditional cell injection media of DMEM, with low oxygen levels, and high levels of oxidative metabolites and inflammatory cytokines, may be responsible for the low survival rate of SCs after implantation [194]. In the interest of graft survival after transplantation, Patel et al. investigated whether transplantation of SCs within various injectable gelling matrixes as suspension cells could improve their long-term survival in the contused SCI model [109]. At the end of the experiment, the matrices which composed of laminin and collagen was reported to show significantly increased capability of improving SC survival, graft vascularization, and axonal in-growth over controls. And the SC transplantation within Matrigel from BD was reported to significantly enhance locomotor function as assessed by the BBB scale. In another study which using the biodegradable polymer scaffolds (polilactic acid and polyglycolic, PLGA), Olson et al. injected SCs within BD Matrigel into the multichannel of the PLGA scaffolds in order to assess the potential of this combined treatment to promote axon regeneration after SCI *in vivo *[110]. One month after transplantation, animals were sacrificed for immunohistochemical staining. This paper showed significant axonal regeneration, but the lack of behavioral improvements.. There were no significant differences in BBB scores between the SCs+scaffold group and the control group in the one month period after surgery.

The cotransplantation approach has also been tested for SCs in SCI research [195]. After obtaining promising results *in vitro*, Ban et al. transplanted SCs and MSCs together into the epicenter of injury [68]. Significantly more regenerated axons in the corticospinal tract, which surrounded and crossed through the posttrauma cavity, were observed in the cograft group. In addition, this group also had the smallest population of GFAP positive astrocytes in the epicenter of injury. Moreover, under electron microscopy, the completely reconstructed myelin sheaths were found in the cograft group. In addition to these histopathological improvements, hindlimb motor function in the cograft group was significantly improved as determined by increased BBB scores. In another co-transplantation experiment, instead of simply mixing different cell types together and injecting the mixture into the animal, Fouad et al. combined the bridging effect of SCs and the MSCs in an innovative way [111]. They seeded SCs into a scaffold with Matrigel, and then implanted this component into the lesion site to build up a bridge for the extension and reconnection of regenerated axons. Then they grafted OECs rostral and caudal to the lesion site, to test whether this approach had any therapeutic advantage. Axonal regeneration within the corticospinal and reticulospinal tracts was not observed through the SCs-scaffold bridge. There was significantly improved motor function as measured by BBB scores and forelimb/hindlimb coupling, which was accompanied by significantly increased numbers of remyelinated serotonergic axons through the bridge.

Not all results from combined treatment experiments have been positive and encouraging. In an *in vivo* experiment, Sharp et al. [112] repeated a previous experiment [196] which exhibited significant locomotor recovery enhancement after contusion injury by means of a combined treatment, which included SC transplantation, systemic delivery of cAMP level enhancer (Rolipram), and intraspinal injection of a non hydrolyzable analog of cAMP (dibutyryl). Almost completely contradictory results were obtained by Sharp et al. compared to the previous study. No significant differences, which include BBB scores, base of support, stride length, and paw rotation were replicated With regards to the anatomical assessments, although a reduction in mean cavity area at the lesion epicenter and remyelinated axons crossing the lesion site were observed in the two groups that received SCs, there were no significant differences between the groups. Bunge and Pearse responded to the replicated work of Sharp, and they provided more details of the original experiment in order to explain the differences [197]. Scott et al. identified some important variables that contribute to explain those different results between the original and replicated study, which included the experimental group arrangements, consistency of injury severity, appropriated statistics, and animal surgery [198]. 

## 9. Clinical Trials

With progress in *in vivo* studies, scientists and surgeons have been eager to conduct clinical trials to explore the therapeutic effects of cell transplantation on spinal cord patients. Various cell types, different administration strategies, and different kinds of SCI patients have been involved in clinical trials, however, several obstacles that are inherent to human studies including ethical issues differences in anatomy, and differences in underlying pathophysiological processes, has hampered progress. Until now, no promising cell therapies that are safe and effective for SCI patients have been achieved.

### 9.1. Clinical Trials of ESC/iPSC or ESC/iPSC-Derived Therapy

The expectation of clinical application by means of an ESC/iPSC or ESC/iPSC-derived therapy has been widely discussed in the media. There are several issues concerning the safety and the efficacy of these stem cell strategies, which may range from target population selection, long-term tumor genesis, to a series of ethical problems [199–201]. According to Aznar and Sánchez, the data available do not justify a clinical trial of stem cell-related therapies, more preclinical study should be carried out and repeated in large animal models of SCI (e.g., cat, dog, rabbit, or primate) [202]. Even though the concern of cyst formation after stem cell transplantation at the injury site was raised by scientists, the Geron Corporation was allowed to run the first clinical trial of stem-cell therapy for SCI in 2009. In the next year, Geron corporation initiated the first clinical trial (Phase I) to test the safety of human embryonic stem cell-derived OPCs, GRNOPC1, within patients who were suffering from complete thoracic level paraplegia with the loss of motor and sensory function [203]. GRNOPC1 was administered into the lesion site within 14 days of injury with a low dose of 2 million cells. To date, there are no serious adverse events in the long-term followup reported by them. Furthermore, they plan to test the safety in patients with a higher cell concentration with 20 million cells in the next step. In November 2011, Geron announced that it had ended its SCI stem cell research program largely due to financial reasons [204]. Based on the work that was completed in the Phase I clinical trial, no therapeutic improvements were reported, although Geron was looking mainly at the safety profile at this stage. So far, no further safety issues have emerged [204]. As this was a significant trial for stem-cell-based therapy for SCI, its premature end, the trial design and the safety results it generated have drawn much attention and interest from researchers around the world. Bretzner et al. proposed a comment to argue the target population selection in the clinical trial of GRNOPC1, and they suggest a more detailed criteria for selecting patients for different study purposes: (1) chronic complete SCI patients for a safety trial, (2) subacute incomplete SCI patients for an efficacy trial, (3) and perhaps primary progressive multiple sclerosis patients for a combined safety and efficacy trial [201]. They posed that the chronic completed SCI patients may be a more preferable target population than subacute complete SCI patients in the phase I clinical trial, because simultaneous recovery may occur in some subacute complete SCI patients and may confound results. In addition, the chronic complete lesion site may ensure a stable microenvironment after cell transplantation in which to assess the safety of transplanted cells [205]. The potential tumorigenicity of ESC-derived OPCs involved in the first clinical trial also concerned scientists, despite several studies reporting the absence of teratomas in rodent experiments [42, 206]. The teratoma-forming propensity was reported to be related with the persistence of undifferentiated cells, even in animal experiments, however, the direct transplantation of undifferentiated ESCs/iPSCs was rare [40]. Du et al. reported a formation of typical teratoma in all immunodeficient experimental mice after the transplantation of hESC-NPCs in spinal cords, but no tumor formation was observed in testis and subcutaneous tissue transplantation group [207]. Teratoma-formation can be suppressed through specific treatments. According to the report from Matsuda et al, the stage-specific embryonic antigen-1 (SSEA-1) expression, which is considered as a marker of teratoma formation, and some mRNA expression markers of undifferentiated ESCs, such as Oct3/4, Utf1, Nanog, Sox2, and Eras, were both significantly reduced in the coculture group of ESCs and bone marrow stromal cells (BMSCs) *in vitro*. The cocultured BMSCs induced undifferentiated ESCs to differentiate into neuronal like MAP-2 positive cells by synthesizing NGF, GDNF, and BDNF *in vitro*. No tumor development was observed after ESCs and BMSCs were grafted together into the mouse SCI model. In contrast, tumor-formation was identified in the solo ESC transplanted group, in which the behavioral improvement also ceased after 21 days of transplantation [208]. In conclusion, ESC/iPSC cell therapies offer promising therapeutic potential for SCI which at this stage waits further clinical testing.

### 9.2. Clinical Trials of Mesenchymal Stem Cells

Although the long-term safety of MSCs therapies has not been well established until now, clinical tests have proceeded. Although the transplantation of MSCs after SCI has shown some promising results in animal experiments, the therapeutic effects of MSC administration in human SCI still remains inefficacious and has had adverse side effects [34, 209, 210]. Ichim et al. reported a MSCs and CD34 cell combined cellular therapy protocol with a total of 13 intrathecal administrations and 2 IV injections in 3 cycles of treatment. In total, 4.05 × 10^7^ CD34 cells and 1.0134 × 10^8^ MSCs were injected into a 29-year old and ASIA scale type A classified patient within a period of 10 month [34]. Sensory function and lower limbs muscle strength recovery was assessed during the procedure, and significant improvements were measured at the end of treatment. The 10/10 pretreatment neuropathic pain was significantly relieved into occasional pain once a week at a level of 3/10. Six months after the end of treatment, this patient was finally categorized as ASIA type D. Moreover, they reported that neither the immunological reactions nor GVHD was noted. However, this case report did not show evidence from biochemical marker analysis or cell-tracking studies to defend their conclusion that the encouraging functional recovery was caused by the effect of grafted cells, and not spontaneously. Intrathecal administration of MSCs for chronic complete SCI patients, a population of 64 completely injured patients (ASIA Scale: A) who had a mean of 3.6 years rehabilitation therapies 3 times weekly was investigated by Kishk et al. [209]. Autologous MSCs were administrated monthly to forty-five patients for 6 months. 12 months after completing the therapy, a series of paralysis grading systems and a questionnaire of bladder and bowel control were used to evaluate the potential therapeutic effects of MSC administration. However, no differences between the MSCs group and control group were found. More importantly, neuropathic pain was observed in twenty-three MSCs-administrated patients. Therefore, the authors concluded that the safety, the side effects and the potential therapeutic effects of MSCs should be carefully studied via preclinical models before launching clinical trials. Shortly after the report of Kishk et al., another clinical trial of MSCs administration for chronic complete SCI patients was reported by Bhanot et al. [210]. After a laminectomy, the autologous MSCs were administered at the lesion site of spinal cord. At the end of followup, only one patient demonstrated improvement in motor function, and other two patients showed inconsistent improvement in pin prick sensation below the level of injury. The outcome of MSCs therapy from this clinical study was therefore not successful. In 2012, Park et al. and Karamouzian et al. reported two clinical trials for spinal cord injury by using MSCs transplantation, even though some improvements were noticed in some patients, the therapeutic effects of MSCs transplantation have not been established in human SCI patients [211, 212]. In the study of Park et al., 10 traumatic cervical SCI patients with severe paralysis were involved (ASIA classification A or B) [211]. MSCs were administered three times during the course of the study. First of all, 8 × 10^6^ autologous MSCs were directly injected into the intradural space, after 4 and 8 weeks, another 5 × 10^6^ MSCs, each time, were injected into the spinal cord above the lesion cavity and into the cavity, respectively. With a 6-month follow-up, the motor power grade of the extremities, magnetic resonance imaging, and electrophysiological recordings were assessed. At the end of this study, improvements of daily living activities, increased motor power of the upper extremities, shrinked lesion cavity size, and electrophysiological improvement were observed in 3 patients. Various partially improvements were observed in the rest of 7 patients. And, they claimed that no permanent complication associated with MSCs transplantation was observed. Karamouzian et al. transplanted autologous MSCs into the cerebrospinal fluid via lumbar puncture for eleven SCI patients with complete thoracic injuries. As they observed, 5 of 11 patients in the MSCs transplantation group and 3 of 20 patients in the control group showed marked function recovery, however, the differences between the two groups were not significant. On the other hand, no adverse reaction and complications in both groups were experienced by patients, which may indicate the safety of intrathecal administration of MSCs in human patients.

### 9.3. Clinical Trials of Schwann cells

Any potential clinical trials of Schwann cells treatments will require addressing a number of questions and concerns. Similar to the ESC-derived OPCs clinical trial, the clinical experiment design must also fulfill the strictest criteria to ensure the safety of grafts and to protect the involved patients from the threat of tumorigenesis and any other serious side effects. Recovery was reported by Xian-Hu et al. within their 6 clinical cases after 5-years followup. The group tested three different transplantation protocols but provided no information on the the distribution of grafted SCs in the body of patients or the tumorigenesis assessment [213]. Furthermore, they provided no criteria for selecting their patient population. On assessment of their methods and results, several important questions arose such as, the ages of patients which range from 7 to 44 years old, the presurgery ASIA evaluations which ranges from A to C, and the transplantation time post-SCI which range from 1 week to 20 months. Importantly, here as in other clinical studies on SCI, the issues of spontaneous recovery and patient heterogeneity (in terms of age, clinical course, and severity) are central to deriving any meaningful information from these results. In another clinical trial report, Saberi et al. transplanted purified SCs which had been acquired from autologous sural nerve into four patients (22–43 years old) who were suffering from stable chronic SCI (28–80 months posttrauma) [214]. Transient paresthesia or increased muscle spasm after transplantation was found in all the four patients. After one year followup, only one patient with incomplete SCI showed some sort of improvement with extensive and continuous rehabilitation. And neither visible positive changes nor negative pathological findings were observed via magnetic resonance imaging. In 2011, Saberi et al. reported another 2-years followup clinical trial for the safety assessment of SCs transplantation therapy [215]. In this study, 33 patients who suffering from completed cervical or thoracic level paraplegia for at least 6 months were enrolled. According to their report, no case of permanent neurological worsening and no severe postoperative complications were found during the following up period. In addition, no new increment in syrinx size and tumor formation was observed via magnetic resonance imaging. To some extent, these reports might be able to suggest the safety of clinical trials for SC therapy, however, more replicable large animal experiments and phase I clinical trials following critical criteria are necessary before large-scale phase II clinical trial can be attempted. 

## 10. *In Vivo* Tracking of Stem Cells

Although the cellular behavior of grafted cells *in vivo*, which include cell survival, migration, and differentiation, can be commonly assessed through various techniques of tissue slice staining, noninvasive real-time observations within living animals or patients are much more useful and informative regarding the fate of these cells *in vivo*. In pace with the development of general magnetic resonance imaging (MRI), cellular MRI techniques that visualize and track grafted cells in living organisms have also expanded considerably in recent years [216, 217]. With the utilization of *in vivo* tracking techniques, scientists can observe the grafted cells directly, and to evaluate important parameters of transplanted cells, such as, the survival, the distribution pattern, the route of migration, and the integration with host tissue [216]. In order to track grafted stem cells in the injured spinal cord by cellular MRI, the stem cells must be labeled with of magnetic particles prior to transplantation. Recently, several kinds of magnetic particles are available for labeling multiple-cell lines, for example, superparamagnetic iron oxide nanoparticles (SPION), magnetic CoPt nanoparticles, Gd-DTPA, and FDA-approved ferumoxytol [218–221].

Among all the label particles, magnetic labeling with SPION is the most widely used and developed method. Scientists have used a variety of improved methods to generate several subtypes of SPION with distinct coating for promoting the uptake rate, extending the effect duration, enhancing the resolution of signal, and decreasing the toxicity. Lee et al. coated SPION with unfractionated heparin (UFH) as a novel negative contrast agent for tracking MSCs *in vivo*. The uptake efficiency of UFH-SPION by MSCs was reported to be improved by threefold when compared with dextran coated SPION. Moreover, no transfection agents were involved to help uptake by MSCs. They suggested that the UFH-SPION uptake was likely mediated by endocytosis, and internalized into the cytosol of MSCs to maintain the visualization for 28 days *in vitro*. After transplantation into nude mice, UFH-SPION remained detectable by T2-weighted MRI for one month [222]. According to Andreas et al., the low-labeling efficiencies and the need of potentially toxic transfection agents are the main obstacles to the utilization of commercial SPION [221]. Because of this reason, they coated SPION with citrate, and then compared the labeling efficiencies effects on stem cell functionality, and the *in vivo* MRI visualization of new citrate-coated SPION with commercial Endorem and Resovist SPION. The citrate-coated SPION presented significantly better uptake efficacy without the presence of transfection agents, and *in vivo* visualization by MRI in the comparison. Although the expression of MSC surface marker antigens and differentiation into the adipogenic and osteogenic lineages were not affected by citrate-coated SPION labeling, the chondrogenic differentiation were significantly impaired with increasing amounts of citrate-coated SPION incorporation [221]. The influence on neurogenic differentiation was however, not assessed. 

Another experimentally coated SPION, chitosan-coated SPION, was verified by Reddy et al. for their labeling efficiency of MSCs. Interestedly, 100% labeling efficiency with no alterations in the surface markers expression and differentiation potential was reported. After transplantation of chitosan-coated SPION labeled MSCs into rabbit ischemic brain, the distribution and migration of labeled MSCs at day 16 was clearly visualized on T2-weighted images and susceptibility weighted images. In addition, the size of the ischemic area was significantly decreased at day 16 when compared with an early time point of day 4 [223].

To observe the fate of transplanted NSCs *in vivo*, Meng et al. labeled the cells with magnetic CoPt hollow nanoparticles (CoPt-NPs) for MRI detection. First the optimized nanoparticle concentration that had no negative impacts on cell viability and the effects on differentiation potential were assessed *in vitro. *In the second step, *ex vivo*, the CoPt-NPs labeled NSCs were transplanted into organotypic spinal cord slices. As they expected, a small number of labeled NSCs could be identified by MRI efficiently with enhanced image contrast [219]. Although iron oxide nanoparticle based tracking of stem cells is effective and reproducible, the intrinsic iron signal derived from erythrocytes may be able to mask target cells *in vivo* [217]. Liu et al. verified the safety and feasibility of applying gadolinium-diethylene triamine pentaacetic acid (Gd-DTPA) for T1W signal enhancement for MSC tracking in a rat SCI model. After obtaining promising results in the differentiation assay *in vitro*, the Gd-DTPA labeled MSCs were transplanted into the lesion site of SCI rat, and the positive signal enhancement of labeled cells on T1W images was detected in the duration from 3 to 14 days. Furthermore, the BBB scores in the Gd-DTPA labeled MSCs transplantation group were significantly higher than the control group [224]. However, the observation period of 14 days was less than the time required to evaluate the long-term effects of transplanted stem cells in the treatment for SCI animal. Additional approaches for long-term assessment of labeled cells were developed by scientists. Berman et al. labeled NSCs with SPION, transfected them with the luciferase bioluminescence reporter gene, and then transplanted these cells into the brains of mice. Over a long-term period of 93 days, the bioluminescence signal was able to be detected via 3D surface topography imaging device. In addition, the hypointensities from the SPIO label in T2W image were also detectable over the course of the experiment [225].

## 11. Conclusion

Taken all above together, in order to achieve the dream of saving the life of SCI patients, improving the life quality and curing the injured spinal cord completely, cellular replacement therapies have recently attracted a lot of attention and several recent publications have shed light on the mechanisms involved and potential hurdles that need to be overcome for the successful translation of this approach. Scientists have been trying all efforts to improve various experimental methods which contribute to the reconstruction of histologically impaired tissue structure, and to the restoration of neural function eventually. With the development of stem cell therapy research, the capability of promoting neuroregeneration of various stem cells is becoming gradually clear, and the obstacles have been overcome one by one. A multipronged approach may be the only effective way to improve functional recovery after SCI. Not all cell populations will have the same effects on each of these modalities, but the population(s) with the maximal effects may have eventual therapeutic benefit for SCI.

## Figures and Tables

**Table 1:** *In vivo* transplantations of ESCs and iPSCs.

First author	Year	SCI Models	Main graft	Transplantation methods	Pre-differentiation or pretreated	Combination	Tissue sparing	Neuronal regeneration	Axonal regeneration /remyelination	Sensory function	Motor function	Cavity and/or scare formation	Inflammation	Others
Bottai, [42]	2010	T8, contusion -mice	Mouse ESCs	i.v.	—	—	—	—	Imp.	—	Imp.	—	Decreased nr. of macrophages and neutrophils	—
Kumagai, [43]	2009	T10, contusion -mice	Mouse ESCs	Lesion epicenter i. medu.	Neurogenic neurospheres	—	Imp.	No Imp.	No Imp.	—	No Imp.	—	—	No angiogenesis
			Mouse ESCs	Lesion epicenter i. medu.	Gliogenic neurospheres	—	Imp.	Imp.	Imp.	—	Imp.	—	—	Enhanced angiogenesis
Lowry, [44]	2008	T8, dorsal hemisection-mice	Mouse ESCs	Lesion site and rostral i. medu.	Endothelial cells/hedgehog /retinoci acid pre-treated	—	—	Imp.	Imp.	Imp.	Imp.	—	—	—
Fujimoto, [45]	2012	T10, contusion -mice	Human iPSCs	Lesion epicenter i. medu.	Neuroepithelial-like stem cells	—	Imp.	Imp.	Imp.	—	Imp.	—	—	—
Chen, [46]	2005	T8, compression -mice	Primed-hNSCs	Rostral and caudal i. medu.	L1-transfection	—	Imp.	Imp.	Imp.	—	—	Imp.	—	—
Cui, [47]	2011	T9, compression -mice	Mouse ESCs	Rostral and caudal i. medu.	Neuronal differentiation and L1 expression	—	Imp.	Imp.	Imp.	—	Imp.	—	Decreased microglial reaction	—
Perrin, [48]	2010	T9, compression -rats	Human ESCs	Lesion site, rostral and caudal i. medu.	Ngn2-transfection	—	No Imp.	Imp.	Imp.	—	Imp.	—	—	—
Hatami, [49]	2009	T10, lateral hemisection-rats	Human ESCs	Lesion site, i. medu.	Differentiated to NPs	Collagen I scaffold	—	Imp.	—	Imp.	Imp.	—	—	—
Niapour, [50]	2012	T9, contusion-rats	Human ESCs	Lesion epicenter i. medu.	Differentiated to NPs	NPs + rat SCs	—	Imp.	Imp.	—	Imp.	—	—	—
Rossi, [51]	2010	C5-C6, contusion -rats	Human ESCs	ventral horn, rostral and caudal i. medu.	Differentiated to MPs	—	—	Imp.	Imp.	—	Imp.	—	—	—
Kim, [52]	2010	T13, lateral hemisection-rats	Mouse ESCs	s. i.	GABAergic differentiation	—	—	—	—	Imp.	—	—	—	Increased evoked activity of WDR neurons
Keirstead, [53]	2005	T10, contusion -rats	Human ESCs	Rostral and caudal i. medu.	OPCs differentiation	—	No Imp.	Imp.	Imp.	—	Imp.	—	—	—
Kerr, [54]	2010	T9, contusion-rats	Human ESCs	Lesion site, i. medu.	OPCs differentiation	—	—	Imp.	Imp.	—	Imp.	Imp.	—	Improved electrophysiological activities
Sharp, [55]	2010	C5, contusion-rats	Human ESCs	Rostral and caudal i. medu.	OPCs differentiation	—	Imp.	—	Imp.	—	Imp.	Imp.	Suppression of acute inflammation	—
Erceg, [56]	2010	T8, complete transection-rats	Human ESCs	Rostral and caudal i. medu.	OPCs and MPs differentiation	OPCs + MPs	—	Imp.	Imp.	—	Imp.	—	—	Improved electrophysiological activities
Salehi, [57]	2009	T9, complete transection-rats	Human ESCs	Lesion site, i. medu.	MNs differentiation	MNs + OECs	Imp.	Imp.	Imp.	—	Imp.	Imp.	—	—

—: Not reported; Imp.: Improvement; i.v.: intravenously; i. medu.: intramedullary injection; s. i.: subarachnoid injection; SCI: spinal cord injury; NPs: neural precursors; OPCs: oligodendrocyte progenitor cells; MPs: motoneuron progenitors; MNs: motoneurons, SCs: Schwann cells; OECs: olfactory ensheathing cells.

**Table 2:** *In vivo* transplantations of mesenchymal stem cells.

First author	Year	SCI Models	Main graft	Transplantation methods	Pre-differentiation or pretreated	Combination	Tissue sparing	Neuronal regeneration	Axonal regeneration /remyelination	Sensory function	Motor function	Cavity and/or scare formation	Inflammation	Others
Nakajima, [58]	2012	T9-T10, contusion-rats	Rats bMSCs	Lesion epicenter i. medu.	—	—	Imp.	—	Imp.	—	Imp.	Imp.	Shifting of macrophage phenotype	—
Karaoz, [59]	2012	T9–T11, contusion-rats	Rats bMSCs	Lesion epicenter i. medu.	—	—	Imp.	—	—	—	Imp.	—	—	Only Nestin+/GFAP+ astrocytic-like cells were observed
Park, [60]	2010	T9, contusion-rats	Rats bMSCs	Lesion epicenter i. medu.	—	—	—	No Imp.	—	—	more rapid restora- tion*	—	Decreased nr. of macrophages and monocytes	No bladder function improvement
Abrams, [61]	2009	T11-12, contusion-rats	Rats bMSCs	Lesion site, rostral and caudal i. medu.	—	—	—	—	—	Partially Imp.	Imp.	Imp.	Attenuated chronic inflammation	—
Kang, [62]	2012	T8–10, contusion-rats	Rats bMSCs	i.v.	—	—	—	No Imp.	—	—	Imp.	—	—	—
		T8–10, contusion-rats	Rats bMSCs	Rostral and caudal i. medu.	—	—	—	NeuN+ differentiation	—	—	Imp.	—	—	Expression of BDNF and NGF
Osaka, [63]	2010	T9, contusion-rats	Rats bMSCs	i.v.	—	—	—	—	—	—	Imp.	Imp.	—	—
Mothe, [64]	2011	T8-9, clip compression-rats	Rats bMSCs	i. thec.	—	—	—	No Imp.	Imp.	—	—	—	—	—
Boido, [65]	2012	T9, compressed-mice	Mice bMSCs	Lesion site, i. medu.	—	—	Imp.	No Imp.	—	Imp.	Imp.	—	—	—
Gu, [66]	2010	T9, contusion-rats	Rats bMSCs	Rostral and caudal i. medu.	—	—	Imp.	No Imp.	Imp.	Imp.	Imp.	Imp.	—	Expression of BDNF and GDNF
Alexanian, [67]	2011	T9, contusion-rats	Human bMSCs	Rostral and caudal i. medu.	Neural differentiation	—	Imp.	Imp.	—	No Imp.	Imp.	Imp.	—	—
Ban, [68]	2011	T9, contusion-rats	Rats bMSCs	Lesion epicenter i. medu.	—	MSCs + SCs	—	—	Imp.	—	Imp.	Imp.	—	—
Cho, [69]	2009	T9, contusion-rats	Rats bMSCs	Lesion epicenter i. medu.	Neural differentiation	—	—	Imp.	—	—	Imp.	—	—	Improvements in SSEPs and MEPs
Pedram, [70]	2010	T8-9, catheter compression-rats	Rats bMSCs	Rostral and caudal i. medu.	Neural differentiation	Differentiated and undifferentiated MSCs	—	Imp.	—	—	Imp.	—	—	—
Liu, [71]	2011	T9, contusion-rats	Rats bMSCs	Lesion epicenter i. medu.	bFGF-transfection	—	—	Imp.	Imp.	—	Imp.	—	—	—
Zhang, [72]	2012	T9, ethidium bromide-induced demyelination-rats	Rats bMSCs	Lesion site, i. medu.	NT-3-transfection	—	—	Imp.	Imp.	—	Imp.	—	—	Improvements in SCEPs
Zeng, [73]	2011	T8, complete transection-rats	Human bMSCs	Lesion site, i. medu.	—	Scaffolds + MSCs	—	Imp.	Imp.	—	Imp.	Imp.	Decreased nr. of macrophages and microglial	Enhanced angiogenesis
Kang, [74]	2012	T8-9, complete removal of a 2-mm length of spinal cord	Human bMSCs	Lesion site, i. medu.	—	Scaffolds + MSCs	—	Imp.	Imp.	—	Imp.	—	—	Improvements in MEPs
Park, [75]	2012	L2-3 balloon catheter compression-dogs	Canine aMSCs	Lesion site, i. medu.	Neural differentiation	Matrigel + neural-induced MSCs	—	Imp.	—	—	Imp.	Imp.	Decreased expression of inflammation markers	Increased expression of neurotrophic markers
Guo, [76]	2011	T9, contusion-rats	Human uMSCs	Lesion epicenter i. medu.	Schwann-like cells	NT-3 + huMSC-derived Schwann-like cells	—	Imp.	—	—	Imp.	—	—	—
Shang, [77]	2011	T9, contusion-rats	Human uMSCs	Lesion epicenter i. medu.	NT-3-transfection	—	—	Imp.	Imp.		Imp.	Imp.	—	—
Lee, [78]	2011	L2-3 balloon catheter compression-dogs	Human uMSCs	Lesion site, rostral and caudal i. medu.	—	—	—	Imp.	Imp.		Imp.	—	—	Expression of BDNF and NT-4

*More rapid restoration of hindlimb function without significant differences compared with control groups; —: Not reported; Imp., Improvement; i.v.: intravenously; i. medu.: intramedullary injection; i. thec.: Intrathecal implantation; SCI: spinal cord injury; SCs: Schwann cells; bMSCs: bone-marrow-derived Mesenchymal stem cells; aMSCs: canine adipose-derived mesenchymal stem cells; uMSCs: umbilical-cord mesenchymal stem cells.

**Table 3:** *In vivo* transplantations of neural stem/progenitor cells.

First author	Year	SCI Models	Main graft	Transplantation methods	Pre-differentiation or pretreated	Combination	Tissue sparing	Neuronal regeneration	Axonal regeneration /remyelination	Sensory function	Motor function	Cavity and/or scare formation	Inflammation	Others
Åkesson, [79]	2007	T8, clamp compression-rats	human spinal cord-derived neurospheres	Lesion epicenter i. medu.	—	—	—	Imp.	—	—	—	—	—	—
Webber, [80]	2007	C4, dorsal hemisection-rats	Fetal NSCs-rats	Lesion site, rostral and caudal i. medu.	—	—	—	No Imp.	No Imp.	Partially Imp.	No Imp.	—	—	High glial differentiation rate
Tarasenko, [81]	2007	T9-10, contusion-rats	Human fetal NSCs	Lesion epicenter i. medu.	—	—	—	Imp.	Imp.	—	Imp.	—	—	—
Yan, [82]	2007	L4 and L5 roots avulsion—nude rats	Human fetal NSCs	Lesion epicenter i. medu.	—	—	—	Imp.	Imp.	—	—	—	—	Synaptic contact reformation
Yasuda, [83]	2011	T10, contusion-mice	Mouse fetal NSCs	Lesion epicenter i. medu.	—	—	Imp.	Imp.	Imp.	—	Imp.	—	—	—
Hwang, [84]	2009	T9-10, contusion-rats	Human fetal NSCs	Lesion epicenter and rostral i. medu.	Olig2-transfection	—	Imp.	Imp.	Imp.	Imp.	Imp.	Imp.	—	—
Alexanian, [85]	2010	T8, compressed-rats	Human fetal NPs	Rostral and caudal i. medu.	Differentiated to OPCs	—	—	—	Imp.	Imp.	Imp.	—	—	—
Wang, [86]	2010	3/4 lateral transection-rats	Rats fetal NSCs	Rostral and caudal i. medu.	—	NSCs + OECs	—	Imp.	Imp.	—	Imp.	—	—	—
Salazar, [87]	2010	T9, contusion-mice	Human fetal NSCs	Rostral and caudal i. medu.	—	—	No Imp.	Imp.	—	No Imp.	Imp.	No Imp.	—	—

—: Not reported; Imp.: Improvement; i. medu.: intramedullary injection; SCI: spinal cord injury; NSCs: neural stem cells; NPs: neural precursors; OPCs: oligodendrocyte progenitor cells; OECs: olfactory ensheathing cells.

**Table 4:** *In vivo* transplantations of olfactory ensheathing cells.

First author	Year	SCI Models	Main graft	Transplantation methods	Pre-differentiation or pretreated	Combination	Tissue sparing	Neuronal regeneration	Axonal regeneration /remyelination	Sensory function	Motor function	Cavity and/or scare formation	Inflammation	Others
Ziegler, [88]	2011	T9, complete transection-rats	Rats OECs	Rostral and caudal i. medu.	—	—	—	—	—	—	Imp.	—	—	Improvements in MEPs
Lu, [89]	2006	C4, dorsal hemisection-rats	Rats OECs	Rostral and caudal i. medu.	—	—	—	No Imp.	Partially Imp.	—	—	—	—	—
Collazos-Castro, [90]	2005	C7, contusion-rats	Rats OECs	Rostral and caudal i. medu.	—	—	—	No Imp.	No Imp.	—	Partially Imp.	—	—	—
Centenaro, [91]	2011	T9, complete transection-rats	OLP and RLP-rats	Lesion site, i. medu.	—	—	—	—	Imp.	—	No Imp.	—	—	—
Aoki, [92]	2010	T10, complete transection-rats	Rats-whole-layer olfactory mucosa	Rostral and caudal i. medu.	—	—	—	—	Partially Imp.	—	Partially Imp.	No Imp.	—	—
Richter, [93]	2005	C4, dorsal lateral hemisection-rats	Mouse lamina propria-derived OECs	Lesion epicenter and rostral i. medu.	—	—	Imp.	—	Imp.	—	—	Imp.	—	Enhanced angiogenesis
Zhang, [94]	2011	T10, contusion-rats	OLP and RLP-rats	Lesion site, i. medu.	—	—	—	—	Imp.	—	—	—	—	Activation of host SCs
Zhang, [95]	2011	T10, contusion-rats	Rats lamina propria-derived OECs	Rostral and caudal i. medu.	—	Glial scar ablation + OECs	—	—	Imp.	—	No Imp.	Imp.	—	Activation of host SCs
Yamamoto, [96]	2009	T10, hemisection-rats	Rats OECs	Lesion epicenter i. medu.	—	—	—	—	No Imp.	—	—	—	—	—
Muñoz-Quiles, [97]	2009	T9, complete transection-rats	Rats OECs	Rostral and caudal i. medu.	—	—	—	—	Imp.		Imp.	Imp.	—	Best results in sub-acute transplantation group
Novikova, [98]	2011	C4, lateral hemisection-rats	Rats OECs	Rostral and caudal i. medu.	3 Weeks or 7 weeks pre-culture	—	—	Imp.	Imp.	—	—	—	—	Aged cells are less effective
Toft, [99]	2007	L3, lateral hemisection-rats	Rats OECs	Lesion site, i. medu.	—	—	—	—	Imp.	—	—	Imp.	—	Improvements in cord dorsum potentials and SEPs
Liu, [100]	2010	T11-12, catheter compression-rats	Rats OECs	Lesion epicenter i. medu.	—	—	—	Imp.	—	—	Imp.	—	—	Improvements in SEPs and MEPs
alinčík, [101]	2010	T4, complete transection-rats	Rats OECs	Lesion epicenter i. medu.	—	—	—	Imp.	—	—	—	—	—	Improvements of autonomic dysreflexia
Tharion, [102]	2011	T10, contusion-rats	Rats OECs	Lesion site, rostral and caudal i. medu.	—	—	—	—	Imp.	—	Imp.	—	—	Improvements of MEPs
Stamegna, [103]	2011	C2, lateral hemisection-rats	Rats OECs	Lesion site, rostral and caudal i. medu.	—	—	—	—	Imp.	—	Imp.	Imp.	—	Improvements in diaphragm activities Improvements in phrenic nerve activities
Bretzner, [104]	2010	C4, dorsal lateral hemisection-rats	Mice OECs	Rostral and caudal i. medu.	—	cAMP infusion + OECs	Imp.	—	Imp.	Imp.	Imp.	Imp.	—	—
Ma, [105]	2010	T9, contusion-rats	Rats OECs	Lesion level, rostral and caudal s. i.	NT-3-transfection	—	—	—	Imp.	—	Imp.	—	—	—
Salehi, [57]	2009	T9, complete transection-rats	Rats OECs	Lesion site, i. medu.	—	MNs + OECs	Imp.	Imp.	Imp.	—	Imp.	Imp.	—	—
Amemori, [106]	2010	T8 balloon catheter compression-rats	Rats OECs	Rostral and caudal i. medu.	—	OECs + MSCs	Imp.	—	—	Partially Imp.	Partially Imp.	—	—	Partial improvements of MEPs

—: Not reported; Imp.: Improvement; i. medu.: intramedullary injection; s. i.: subarachnoid injection; SCI: spinal cord injury; MNs: motoneurons, SCs: Schwann cells; OECs: olfactory ensheathing cells; RLP: respiratory lamina propria; OLP: olfactory lamina propria; MSCs: mesenchymal stem cells.

**Table 5:** *In vivo* transplantations of Schwann cells.

First author	Year	SCI Models	Main graft	Transplantation methods	Pre-differentiation or pretreated	Combination	Tissue sparing	Neuronal regeneration	Axonal regeneration /remyelination	Sensory function	Motor function	Cavity and/or scare formation	Inflammation	Others
Biernaskie, [107]	2007	T10, contusion-rats	Rats SKP-SCs	Lesion epicenter i. medu.	—	—	Imp.	Imp.	Imp.	—	Imp.	Imp.	—	—
Agudo, [108]	2008	C4, dorsal hemisection-rats	SCPs-postnatal 2d rats	Rostral and caudal i. medu.	—	—	—	—	Imp.	No Imp.	No Imp.	Imp.	—	Enhanced angiogenesis
Patel, [109]	2010	T9, contusion-rats	Rats SCs	Lesion epicenter i. medu.	—	Gelling matrix (laminin + collagen) + SCs	—	—	Imp.	—	Imp.	—	—	—
Olson, [110]	2009	T8-9, transection-rats	Rats SCs	Lesion epicenter i. medu.	—	Gelling matrix + NSCs + SCs	—	Imp.	Imp.	—	No Imp.	—	—	—
Ban, [68]	2011	T9, contusion-rats	Rats SCs	Lesion epicenter i. medu.	—	MSCs + SCs	—	—	Imp.	—	Imp.	Imp.	—	—
Fouad, [111]	2005	T8, complete removal of a 4-mm length of spinal cord-rats	Rats SCs	Lesion site, rostral and caudal i. medu.	—	Gelling matrix + MSCs + SCs	—	—	Imp.	Imp.	Imp.	—	—	—
Sharp, [112]	2012	T9, contusion-rats	Rats SCs	Lesion site, i. medu.	—	SCs + dbCAMP	—	—	Imp.	—	No Imp.	—	—	—

—: Not reported; Imp.: Improvement; i. medu.: intramedullary injection; SCI: spinal cord injury; SCs: Schwann cells; SKP: skin-derived precursors; SCPs: Schwann cell precursors; dbCAMP, dibutyryl CAMP; MSCs: mesenchymal stem cells.
